# Dysregulated Expression of Canonical and Non-Canonical Glycolytic Enzyme Isoforms in Peripheral Blood from Subjects with Alcohol Use Disorder and from Individuals with Acute Alcohol Consumption

**DOI:** 10.3390/antiox14091143

**Published:** 2025-09-22

**Authors:** Maura Rojas-Pirela, Daniel Salete-Granado, Diego Andrade-Alviárez, Alejandro Prieto-Rojas, Cristina Rodríguez, María-Lourdes Aguilar-Sánchez, David Puertas-Miranda, María-Ángeles Pérez-Nieto, Vanessa Rueda-Cala, Candy Pérez, Wilfredo Quiñones, Paul A. M. Michels, Ángeles Almeida, Miguel Marcos

**Affiliations:** 1 Instituto de Investigación Biomédica de Salamanca (IBSAL), 37007 Salamanca, Spain; 2 Departamento de Medicina, Universidad de Salamanca, 37007 Salamanca, Spain; 3 Servicio de Medicina Interna, Hospital Universitario de Salamanca, 37007 Salamanca, Spain; 4 Laboratorio de Enzimología de Parásitos, Departamento de Biología, Facultad de Ciencias, Universidad de Los Andes, Mérida 5101, Venezuela; 5 Facultad de Medicina, Universidad de Los Andes, Mérida 5101, Venezuela; 6 Instituto de Biología Funcional y Genómica (IBFG), Consejo Superior de Investigaciones Científicas (CSIC), Universidad de Salamanca, 37007 Salamanca, Spain; 7 Servicio de Psiquiatría, Hospital Universitario de Salamanca, 37007 Salamanca, Spain; 8 Fundación Instituto de Estudios de Ciencias de la Salud de Castilla y León, 42002 Soria, Spain; 9 School of Biological Sciences, University of Edinburgh, The King’s Buildings, Edinburgh EH9 3FL, UK

**Keywords:** Glycolysis, metabolism, alcohol, diseases, altered expression

## Abstract

Glycolysis is primarily involved in ATP production but also modulates oxidative stress. Chronic alcohol consumption is correlated with an increased incidence of multiple diseases, including cancer and neurodegenerative diseases (NDDs), though the underlying mechanisms remain unclear. Guided by a literature review and bioinformatics analysis, we evaluated the expression of 22 genes encoding various isoforms of seven glycolytic enzymes (GEs) in the peripheral blood of patients with alcohol use disorder (AUD), individuals with acute alcohol consumption (AAC), and their respective control groups using qPCR. In parallel, we evaluated the expression of selected genes coding for GEs linked to NDDs, as well as astrocytic markers in primary mouse astrocyte cultures exposed to ethanol. Thirteen GE-related genes, including non-canonical isoforms, were significantly dysregulated in AUD patients; notably, eight of these genes showed similar alterations in individuals with AAC. Several enzymes encoded by these genes are known to be regulated by oxidative stress. Ethanol-exposed astrocytes also showed altered expression of glycolytic genes associated with NDDs and astrocyte function. These findings indicate that glycolytic dysregulation is driven by ethanol intake, regardless of exposure duration or organic damage, highlighting a link between ethanol-driven redox imbalance and glycolytic remodeling, which could contribute to organ damage.

## 1. Introduction

Alcohol consumption is a major public health concern, linked to high morbidity, mortality, and healthcare burdens. Alcohol use disorder (AUD) is a highly prevalent condition often accompanied by comorbidities and disabling symptoms [1]. In 2019, alcohol consumption accounted for approximately 2.6 million deaths worldwide, 1.6 million of which were due to non-communicable diseases, including liver disease and cancer [2]. Notably, the World Health Organization has confirmed that alcohol is a proven carcinogen, with no safe level of intake. Approximately 11–12% of all cancers are directly attributable to alcohol consumption [2,3], but the precise mechanisms underlying this association are not yet fully understood.

The damage caused by alcohol intake is associated with immune activation and metabolic alterations, largely driven by oxidative stress and the production of reactive metabolites [4]. In this context, some studies support the idea that alcohol also dysregulates other major metabolic pathways, such as glycolysis [5]. Indeed, chronic alcohol consumption disrupts glucose metabolism, leading to energy imbalance and accumulation of metabolic by-products that may contribute to ethanol-related inflammation and cancer [6]. These metabolic derangements, together with sustained oxidative stress and defective autophagy, create a vicious cycle that accelerates tissue damage [7]. Importantly, oxidative stress is not only a consequence of ethanol metabolism but also a modulator of glycolytic activity, establishing a mechanistic link between redox imbalance and altered energy metabolism.

Ethanol-related alterations in glycolysis have been previously linked to alcohol-related liver disease (ARLD): in patients with alcoholic hepatitis (AH) and alcohol-associated cirrhosis (AAC), upregulation of specific hexokinase (HK) isoenzymes correlates with acute kidney injury and reduced survival [8], while enolase (ENO) activity in blood cells has been proposed as a marker of ARLD [9]. In contrast, the impact of ethanol on glycolysis in the brain has not been explored, even though chronic alcohol consumption is associated with alcohol-related brain damage (ARBD). This condition is characterized by neurocognitive impairment due to structural and functional alterations, including abnormalities in glucose metabolism. Up to 80% of ARBD cases may progress to severe and irreversible neurological disorders, and some data suggest an overlap between ARBD and neurodegenerative diseases (NDDs), such as Alzheimer’s disease and early-onset dementia [10]. Together, these observations highlight the need for further research into glycolytic disruption as a driver of ethanol-induced brain injury.

**Figure 1:**
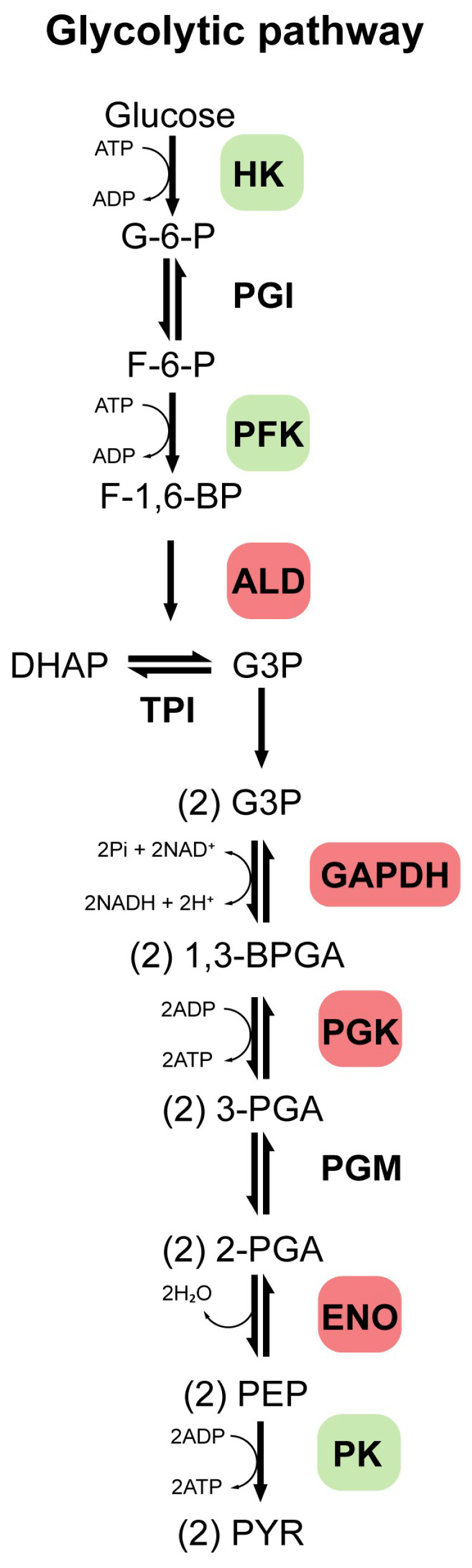
Glycolytic pathway. This pathway comprises 10 enzymatically catalyzed steps, with regulation primarily occurring through the enzymes hexokinase (HK), phosphofructokinase (PFK), and pyruvate kinase (PK), which catalyze reactions that are essentially irreversible under physiological conditions. Additionally, other enzymes such as aldolase (ALD), glyceraldehyde-3-phosphate dehydrogenase (GAPDH), phosphoglycerate kinase (PGK), and enolase (ENO), are also highlighted, as they may play essential roles in glycolytic regulation and are associated with the development of various diseases. Other enzymes such as phosphoglucose isomerase (PGI), triosephosphate isomerase (TPI), and phosphoglycerate mutase (PGM) are also involved in this pathway. A series of intermediate compounds is formed as glucose is progressively broken down into pyruvate. These intermediates are successively: glucose 6-phosphate (G-6-P), fructose 6-phosphate (F-6-P), fructose 1,6-bisphosphate (F-1,6-BP), dihydroxyacetone phosphate (DHAP), glyceraldehyde 3-phosphate (G-3-P), 1,3-bisphosphoglycerate (1,3-BPGA), 3-phosphoglycerate (3-PGA), 2-phosphoglycerate (2-PGA), phosphoenolpyruvate (PEP), pyruvate (PYR).

Glycolysis is a ubiquitous, highly conserved metabolic pathway that catabolizes glucose to pyruvate, generating ATP, NADH, and biosynthetic precursors. It comprises ten enzyme-catalyzed steps (Figure 1), with key regulation exerted by HK, phosphofructokinase (PFK), and pyruvate kinase (PK), which catalyze essentially irreversible reactions under physiological conditions [11]. In humans, GEs exist as isoforms with tissue-specific and developmentally regulated expression patterns [11]. Dysregulation of key GEs (such as HK, PFK, and PK) is closely associated with tumor development, which is often mediated through the Warburg effect (a shift from mitochondrial respiration to aerobic glycolysis), and is also implicated in chronic inflammation, metabolic diseases, and NDDs [11,12,13]. Isoforms of enzymes such as aldolase (ALD), glyceraldehyde-3-phosphate dehydrogenase (GAPDH), phosphoglycerate kinase (PGK), and ENO also play roles in pathological processes [14,15,16]. Beyond metabolism, many GEs perform moonlighting functions in proliferation, transcription, and signaling, particularly in tumor and immune cells [14].

Several GEs, including HK, PFK, ALD, GAPDH, PGK, and PK, have been reported to show altered expression under conditions of redox imbalance as cells attempt to compensate for oxidative stress [17]. Additionally, some of these GEs are redox-sensitive and can undergo transcriptional and/or post-translational modifications under oxidative stress, which can modulate their biological function, even affecting the distribution of the carbon flux between the glycolytic and other pathways, such as the pentose-phosphate pathway [17,18]. Their dysregulation can exacerbate mitochondrial dysfunction and intensify reactive oxygen species (ROS) production, creating a feedforward loop of oxidative damage, particularly relevant in contexts such as NDDs, cancer, and cellular aging [19].

Although changes in glycolysis in peripheral cells do not faithfully mirror those in individual tissues, alterations in peripheral GE activity have been reported in some diseases, such as NDDs and diabetes, highlighting their potential as non-invasive disease biomarkers [20,21]. This is particularly attractive in alcohol related diseases since there are no biomarkers available to assess metabolic alterations and associated organ damage in AUD, nor to stratify patients into optimal treatment groups or predict therapeutic response. This limitation may be at least partly due to the use of models that do not accurately reflect human metabolic changes [22]. Therefore, identifying prognostic biomarkers linked to metabolic pathways capable of objectively and non-invasively monitoring clinically relevant alterations may improve the management of ethanol-related diseases such as ARBD and ARLD. In this context, gene expression profiling should focus on easily accessible cells, such as from peripheral blood (PB) samples, which allow for repeated assessments in the same individual over time [23].

Against this background, although metabolic alterations associated with chronic alcohol use are well recognized in some organs, there is currently a lack of non-invasive biomarkers that can reflect these changes. Furthermore, while glycolytic dysregulation has been described in diseases such as cancer and NDDs, its role in alcohol-related conditions and its detectability in PB cells have been scarcely investigated. Our study addresses this gap by demonstrating that (i) transcriptional rewiring of multiple GEs occurs in the PB of AUD and AAC patients, and (ii) a similar pattern is reproduced in ethanol-exposed primary astrocytes, thus linking systemic and brain-specific metabolic stress. These results suggest a novel, easily accessible, non-invasive peripheral molecular signature that may indicate dysregulated glycolysis and associated metabolic stress, providing a foundation for future studies to explore its potential as a biomarker in alcohol-related diseases.

Therefore, we examined the transcriptional profile of 22 glycolytic-enzyme genes in PB from patients with AUD and matched controls, and we explored whether the same alcohol-related changes appear in primary mouse astrocytes exposed to ethanol. In blood, AUD was associated with marked up-regulation of several canonical and non-canonical isoforms of HK, PFK, ALD, GAPDH, PGK, and PK, isoenzymes previously linked to cancer, inflammation, and liver injury. Overall, HK2, HKDC1, PKM2, ALDA, ALDC, GAPDHS, and PGK isoforms constitute a PB glycolytic signature. Notably, simultaneous overexpression of several non-canonical glycolytic isoforms (HK2, HK3, HKDC1, ALDA, GAPDHS, and PGK2), previously linked to tumor development and progression, has been observed in patients with AUD. Astrocytes showed a concordant dysregulation pattern, including glycolytic genes implicated in neurodegenerative disorders, and altered expression of structural markers such as glial fibrillary acidic protein (GFAP). Taken together, these data suggest that alcohol-induced rewiring of glycolysis is detectable in easily accessible peripheral cells and mirrors metabolic stress in brain cells, supporting its potential as a non-invasive biomarker and mechanistic pathway in alcohol-related disease.

## 2. Materials and Methods

### 2.1. Bioinformatics Studies, Literature Review, and Gene Selection

The bioinformatic analysis of genes of human GEs was conducted through a systematic approach to identify, annotate, and characterize their sequences. Initially, GE gene sequences were retrieved from the National Center for Biotechnology Information (NCBI) database. The presence and verification of catalytic domains within these sequences were assessed using multiple bioinformatics tools and servers, including Protein BLAST (NCBI), the Conserved Domain Database (CDD) [24], InterProScan (EMBL-EBI) [25], and the Simple Modular Architecture Research Tool (SMART) [26], with e-values ranging from 0.0 to 5 × 10^−13^, ensuring high-confidence domain identification. The subcellular localization of the identified GEs was determined based on literature-reported data and predictions obtained from specialized computational tools, namely WoLF PSORT [27], DeepLoc [28], Cell-Ploc [29] and from the Human Protein Atlas (HPA) [30]. Additionally, tissue-specific expression patterns were analyzed to identify isoforms with restricted or predominant expression in particular tissues, thereby determining the biological relevance of their inclusion. The Enrichr bioinformatics platform [31] and bibliographic review were used to explore the relationship between glycolytic genes and neurodegenerative disorders, and alcohol-related pathologies.

### 2.2. Subjects

#### 2.2.1. Sample of Patients with Alcohol Use Disorders (AUD)

Our study included 11 patients with AUD according to the DSM-5 criteria [32], who were referred to the Alcoholism Unit of the University Hospital of Salamanca (Spain). All patients included in this group had a pattern of chronic daily heavy ethanol intake, actively drinking ≥100 g ethanol/day for at least 5 years before they entered the study. All patients had normal prothrombin time, hemoglobin concentration, and serum albumin levels, and they were negative for hepatitis B surface antigen and antibodies to the hepatitis C virus. They did not have other chronic or acute conditions that could interfere with the results of the study, nor were they polydrug abusers. Advanced liver disease was excluded based on clinical, analytical, and ultrasonographic studies: individuals displaying physical stigmata of chronic liver disease (e.g., cutaneous signs, hepatosplenomegaly, gynecomastia, testicular atrophy, and/or muscle wasting) with liver ultrasonographic findings other than steatosis or with increased liver transaminases >2–3 times the reference limits were excluded. In addition, 7 age- and sex-matched healthy volunteers who were reported to drink <15 g of ethanol per day, with normal results in liver function tests and standard hematologic and biochemical tests, were analyzed. Before entering the study, all participants provided informed consent, and the study received approval from the Ethics Committee of the University Hospital of Salamanca (PI2023/07/1389).

#### 2.2.2. Samples from Patients with Acute Alcohol Consumption (AAC)

This clinical study sample included 7 adolescents and young adults (6 male/1 female) who were admitted to the Emergency Department of the University Hospital of Salamanca (Spain) with moderate to severe acute alcohol intoxication, as previously reported [33], with a mean age of 18.71 years (SD = 3.37). Acute alcohol intoxication was defined by clinical signs (e.g., ataxia, slurred speech, impaired reasoning, or disorientation), blood alcohol levels >1 g/L, and the consumption of at least 5 standard drinks (50 g) in men or 4 (40 g) in women during the 6 h before hospital admission. In addition, 7 healthy controls (4 males/3 females) were included in the study, recruited among medical and nursing students, with an average age of 22.57 years (SD = 2.37). Controls did not consume alcohol beyond light sporadic intake, did not drink alcohol during the 72 h before blood draw, and had no binge drinking episodes in the past 3 months. These control subjects displayed normal hematological and plasma biochemical parameters and reported no chronic or acute illness. Urine toxicology screening was performed, and patients whose clinical data or history indicated illicit drug use (except cannabis) were excluded from the study. Other exclusion criteria included chronic or acute illness and medication use. Blood samples were obtained from patients upon admission for standard care and, once patients were no longer under the influence of alcohol and fully capable of understanding the study details, written informed consent was obtained for their use in research.

Although the sample size is relatively small, it is consistent with previous studies that have successfully identified transcriptional changes and potential biomarkers in peripheral blood cells in patients with AUD or alcohol-related conditions [34,35,36].

### 2.3. Sample Processing

Blood samples for hematological and biochemical tests, as well as PAXgene Blood RNA tubes, were obtained from each subject between 8:00 and 10:00 AM under fasting conditions. PAXgene Blood RNA tubes were used to extract total RNA for PCR expression analyses [37].

### 2.4. In Vitro Cultures of Mouse Astrocytes Exposed to Alcohol

#### 2.4.1. Mice

All experimental procedures on animals were performed in accordance with Spanish legislation (RD53/2013) under license from the Spanish government and the European Union (2010/63/EU). Additionally, protocols were approved by the Bioethics Committee of the University of Salamanca. Efforts were made to minimize both animal use and suffering. C57BL/6J mice were used in this study. Animals were maintained in specific-pathogen-free facilities at the University of Salamanca under controlled conditions: a 12 h light/dark cycle, relative humidity of 45–65%, and a temperature range of 20–25 °C. Mice were fed ad libitum with a standard solid diet and had free access to water.

#### 2.4.2. Collection and Primary Culture of Astrocytes

Primary cultures of mouse cortical astrocytes were obtained from newborn C57BL/6J male and female mice on postnatal day P0-1, following the previously described protocol [38]. The cortical tissue was digested with EBSS solution (116 mM NaCl, 5.4 mM KCl, 1.01 mM NaH_2_PO_4_, 1.5 mM MgSO_4,_ 26 mM NaHCO_3 _, 4 mM D-glucose, 10 mg/mL phenol red) supplemented with BSA 0.2% (*w*/*v *), 20 µg/mL DNAse I, and trypsin 0.05% (*w*/*v*). The cellular pellet was resuspended in DMEM low-glucose medium supplemented with 4 mM glutamine, fetal bovine serum 10% (*v*/ *v*), and antibiotics (100 U/mL penicillin, 100 µg/mL streptomycin, and 0.25 µg/mL amphotericin B), seeded in 175 cm^2^ sterile flasks, and incubated at 37 °C with 5% CO_2_. After 7 days in vitro, flasks were shaken overnight at 200 rpm to eliminate non-astrocytic cells. The supernatant was discarded, and the attached astrocyte-enriched cells were reseeded at 50.000 cells/cm^2^ in appropriate plates and maintained for an additional 3 –4 days before use in experiments. The cell cultures were then incubated for 24 h at 37 ° C under the following conditions: controls and medium supplemented with 50 mM ethanol. After the incubation period, the culture medium was discarded, and the cells were washed with PBS. Afterward, 300 μL of TRIzol™ Reagent was added to the plates of cell cultures exposed to different experimental conditions. The lysate was pipetted up and down several times to homogenize. Homogenates were then stored at −80 °C until RNA extraction.

### 2.5. RNA Extraction, Reverse Transcription, and Real-Time PCR (RT-PCR)

The expression of specific mRNAs in PB was determined from the PAXgene™ Blood RNA Tube. To do this, total RNA was first extracted from these tubes using an extraction kit (PAXgene™ Blood RNA Kit), following the manufacturer’s instructions (QIAGEN) and stored at −80 °C. In experiments with astrocytes, the cells were submerged in RNA stabilizing solution (RNAlater, Ambion, Austin, TX, USA) and stored at −80 °C until RNA extraction, which was performed using Trizol reagent (Invitrogen, Thermo Fisher Scientific, Carlsbad, CA, USA) according to the manufacturer’s protocol. RNA extraction was performed with isopropanol/chloroform/ethanol (0.2/0.5/1 mL per 1 mL of Trizol). For all samples, RNA quantity and purity were also examined using Nano-Drop. cDNA was then synthesized from the extracted RNA, using a commercial kit (High-Capacity cDNA Reverse Transcription Kit, Applied Biosystems, Waltham, MA, USA) The mRNA expression levels were determined by the relative quantitative real-time polymerase chain reaction (qPCR), performed in on a StepOnePlus™ Real-Time PCR System (Applied Biosystems) after the primer specificity (Supplementary Table S1) having been verified by melt curve analysis. All qPCR analyses were performed with technical replicates, and the values used for statistical comparisons represent independent biological replicates. The expected length of the PCR products was monitored by gel electrophoresis (2% agarose gel, Bioline, London, UK). Nucleotide sequences of the primer pairs are shown in Supplementary Table S1. Gene 18S ribosomal RNA (18S) or TATA-binding protein (TBP) was used as endogenous control. The threshold cycle (Ct; the number of cycles to reach a threshold of detection) was determined for each reaction, and gene expression was quantified using the 2−ΔΔCt method. Data were normalized and presented as the ratio of their control values.

Heatmaps were generated to visualize glycolytic gene expression profiles in patients with AUD and AAC from qPCR data. Normalized expression levels were transformed using the formula log_2_(X + 1), where X represents the normalized expression value. Heatmaps were constructed using GraphPad Prism (version 8.0.2, GraphPad Software, San Diego, CA, USA).

### 2.6. Statistical Analysis

In Table 1 and Table 2, quantitative variables were expressed as the mean (standard deviation [SD]), while qualitative variables were expressed as the absolute (n) and relative (%) frequencies. In the analysis of qPCR, all data are presented as the mean (standard error of the mean [SEM]). The Ct values were normalized to a reference gene, and statistical significance of the differences in Ct values between control and patient groups, or between control and alcohol-treated cell cultures, was assessed using the Mann–Whitney U test, and effect sizes were calculated using Cliff’s Delta (Cliff’s δ). Differences were considered statistically significant at *p* < 0.05. All statistical analyses were performed using GraphPad Prism software, and specific statistical details are provided in the figure legends.

Multiple comparisons were addressed using the Bonferroni correction in two complementary ways. First, a global adjustment (BfG) was applied considering all genes together (n = 19), with a significance threshold of α = 0.05/19 ≈ 0.0026. Second, a family-wise adjustment (BfF) was applied within each functional gene family, with α = 0.05 divided by the number of genes in the respective family. Raw *p*-values (p_raw) were obtained using the Mann–Whitney U test. Changes in gene expression were considered significant after global adjustment if p_BfG < α and significant after family-wise adjustment if p_BfF< α.

## 3. Results

### 3.1. Bioinformatics Studies, Literature Review, and Gene Selection

Bioinformatics analysis of the genes encoding the three key regulated enzymes of the glycolytic pathway (HK, PFK, and PK) as well as the genes for ALD, GAPDH, PGK, and ENO revealed genetic and functional characteristics associated with disease development (Supplementary Table S2). These enzymes are expressed as different isoforms and either have ubiquitous expression or may be restricted to certain tissues/cells, developmental states, or pathological conditions. In addition to cytosolic localization, many of these enzymes can occupy diverse subcellular compartments, including the mitochondria, with some isoforms reported in up to five distinct locations. Of note, PGK2 is almost exclusively expressed in germ cells, whereas HK2 overexpression is considered a hallmark of tumor cells (Supplementary Table S2). A relevant feature of these glycolytic genes is that they can give rise to one or multiple transcript variants of varying lengths. Many GE isoforms are implicated in diseases associated with alcohol consumption, including various types of cancers, diabetes, inflammation, and liver diseases. Additionally, some have even been proposed as biomarkers or therapeutic targets for conditions such as AH and liver cancer. Some enzymes, such as HK2, ALDC, GAPDH, and ENO2, have been linked to brain injury and NDDs (Supplementary Table S2). Notably, isoforms ENO4 and ENO5, which have been little studied until now, contain non-catalytic domains. Specifically, a dimerization/docking (D/D) domain was identified in ENO4, while ENO5 possesses a capping domain (CD) associated with functions linked to enzymatic activity regulation, structural stability, and protein–protein interactions (Supplementary Tables S2 and S3). To our knowledge, there has been no reference to this domain in ENO5. These structural distinctions could provide new insights into their involvement in metabolic adaptation and disease processes.

Altogether, this comprehensive bioinformatics analysis and literature review provided a rational and evidence-based basis for the guided selection of 22 target GE genes, including canonical and non-canonical isoforms (Supplementary Table S1), for subsequent qPCR expression studies.

### 3.2. Characteristics of the Study Cohort

#### 3.2.1. Patients with AUD

The principal demographic and clinical characteristics of both the patient cohort and the healthy control group are outlined in Table 1. No statistically significant differences were observed between the groups in terms of age and sex distribution. In the patient group, the mean daily alcohol consumption was 146 g (SD = 12.00), with a chronic intake history of at least five years. Biochemical analyses showed significantly elevated serum levels of aspartate aminotransferase (AST), alanine aminotransferase (ALT), and alkaline phosphatase (ALP). Additionally, patients exhibited a significant increase in mean total leukocyte and neutrophil counts, compared to their control counterparts (Table 1).

#### 3.2.2. Patients with AAC

The principal demographic and clinical characteristics of the acute alcohol consumption (AAC) group and the healthy controls are summarized in Table 2. Although there were no statistically significant differences in sex distribution between the groups, AAC patients were significantly younger than controls. Biochemical analyses revealed that among the liver function markers assessed, only AST levels were significantly elevated in AAC patients compared to their control counterparts. Additionally, AAC patients exhibited a significant elevation in total leukocyte count.

### 3.3. Expression of GEs in PB of Patients with AUD

**Figure 2:**
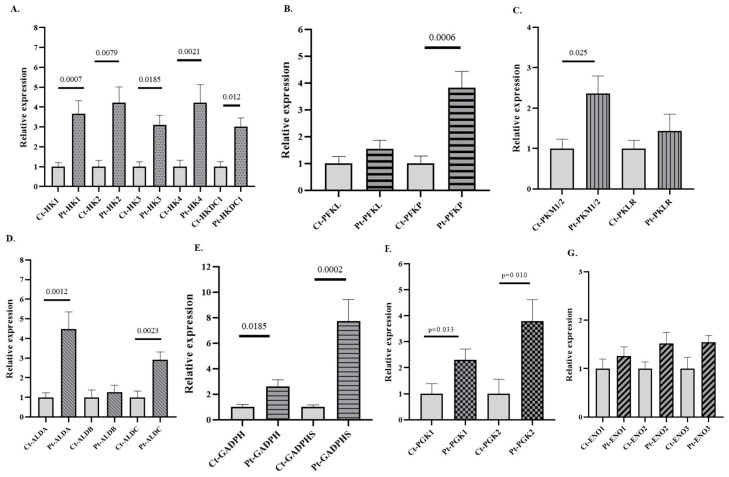
Relative mRNA expression of genes encoding GE isoforms in the peripheral blood (PB) of patients with alcohol use disorder (AUD) and healthy controls. (**A**) Genes encoding hexokinase (HK) isoforms, (**B**) phosphofructokinase (PFK) isoforms, (**C**) pyruvate kinase (PK) isoforms, (**D**) aldolase (ALD) isoforms, (**E**) glyceraldehyde-3-phosphate dehydrogenase (GAPDH) isoforms, (**F**) phosphoglycerate kinase (PGK) isoforms, and (**G**) enolase (ENO) isoforms. Data are presented as relative expression compared to the control group (Data are mean ± SEM). Differences between groups were assessed using the Mann–Whitney U test. Exact *p*-values are shown in the figure. Control group n = 7, AUD group n = 11.

After comparing by qPCR the expression of several isoforms of GEs (HK, PFK, ALD, GAPDH, PGK, ENO, and PK) in PB samples from patients with AUD versus healthy subjects, a significant increase in the expression of all HK, PFK, and PK isoforms was observed in patients with AUD (Figure 2). Notably, all isoforms of GAPDH and PGK were upregulated in patients, but only the ALDA and ALDC isoforms of ALD were overexpressed in the AUD patients. mRNA levels of the canonical isoforms of ENO (ENO1, ENO2, and ENO3) remained unchanged, whereas ENO4 and ENO5 were not detected. Furthermore, differences were observed in the expression of several non-canonical and/or embryonic isoforms (HK3, HKDC1, ALDA, GADPH-S, PGK2, and PKM) between patients and controls, as well as in the expression of isoforms considered hallmarks of cancer, such as HK2 (Figure 2). The expression of PFKM was undetectable in these samples. Although this study is exploratory, we applied Bonferroni correction both globally (BfG) and by gene family (BfF), and the main results remained consistent with the unadjusted analyses. Data about the expression of genes such as HK1, HK4, HKDC1, PFKP, ALDA, ALDC, and GAPDHS remained significant after global adjustment (BG), whereas those for other genes, such as HK2, GADPH, and PGK2, were significant only after BfF (Supplementary Table S5). Cliff’s δ values further supported our observations, as many of the data on the expression of genes that reached statistical significance showed δ values approaching 1. Such high effect sizes are indicative of strong group separation and point toward a potential biological relevance of these transcriptional changes (Supplementary Table S4). Importantly, the expression patterns observed in the graphs were consistently mirrored when presented together in the heatmap, which provides a more visual representation of the data (Supplementary Figure S1).

### 3.4. Expression of GEs in PB of Patients with AAC

**Figure 3:**
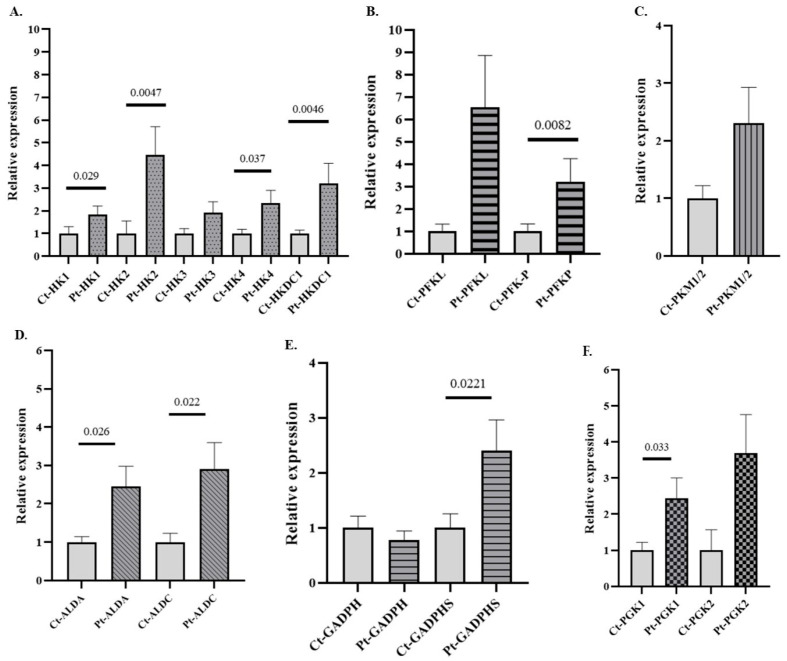
Relative mRNA expression of genes encoding GE isoforms in the peripheral blood (PB) of individuals with acute alcohol consumption (AAC) and healthy controls. (**A**) Genes encoding hexokinase (HK) isoforms, (**B**) phosphofructokinase (PFK) isoforms, (**C**) pyruvate kinase (PK) isoforms, (**D**) aldolase (ALD) isoforms, (**E**) glyceraldehyde-3-phosphate dehydrogenase (GAPDH) isoforms, and (**F**) phosphoglycerate kinase (PGK) isoforms. Data are presented as relative expression compared to the control group (Data are mean ± SEM). Differences between groups were assessed using the Mann–Whitney U test. Exact *p*-values are shown in the figure. Control group n = 7, AAC group n = 7.

To determine whether changes in GE gene expression could also be observed following acute alcohol exposure, we analyzed the gene expression profiles of those genes differentially expressed in AUD patients (see Figure 2) in an independent cohort of individuals with AAC. The results demonstrate that 8 of the 13 differentially expressed glycolytic genes identified in the AUD group also showed significant alterations in the AAC cohort (Figure 3). Notably, genes encoding key regulatory enzymes of the glycolytic pathway, such as HK and PFKP isoforms, as well as non-canonical variants such as HK2, HKDC1, ALDOA, and GAPDHS, were significantly upregulated in individuals with AAC. These results suggest a shared transcriptional response to ethanol, regardless of the duration of exposure or the presence of organic damage. Notably, in these patients, only expression data for three genes (ALDA, ALDC, and GAPDHS) remained significant after global Bonferroni correction (BfG). However, the remaining genes that were predicted to be significant in the unadjusted analysis also remained significant after BfF (Supplementary Table S6). The genes that reached statistical significance also showed Cliff’s δ values close to 1, indicating their potential biological relevance (Supplementary Table S4). The patterns of expression of the glycolytic genes in patients with AAC observed in the graphs are consistently mirrored when presented together in a heatmap, which provides a clearer graphical representation of the data (Supplementary Figure S2)

### 3.5. Expression of GEs in In Vitro Cultures of Mouse Astrocytes Exposed to Alcohol

**Figure 4:**
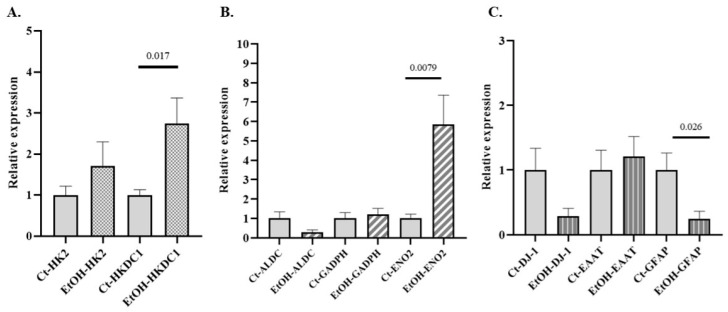
Relative mRNA expression of selected genes in primary mouse astrocyte cultures exposed to ethanol. (**A**) Relative expression of genes encoding isoforms of GEs associated with NDD: (**A**) HK2 and HKDC1 (hexokinase isoforms) and (**B**) aldolase isoform C (ALDC)glyceraldehyde-3-phosphate dehydrogenase (GADPH), neuron-specific enolase isoform 2 (ENO2). (**C**) Relative expression of genes related to astrocytic identity and functionality: glial fibrillary acidic protein (GFAP), parkinsonism-associated deglycase (DJ-1), and excitatory amino acid transporter (EAAT). Data are presented as relative expression compared to the control group (Data are mean ± SEM). Differences between groups were assessed using the Mann–Whitney U test. Exact *p*-values are shown in the figure. Control group n = 6; ethanol-treated group n = 6.

Alterations in glycolytic metabolism have been implicated in the development of several NDDs. The expression of various GE isoforms associated with NDDs (HK2, HKDC1, ALDC, GAPDH, and ENO2 (Supplementary Table S2), along with genes related to astrocytic identity and functionality, such as GFAP, excitatory amino acid transporter (EAAT), and parkinsonism-associated deglycase (DJ-1), was evaluated in primary cultures of astrocytes exposed to alcohol, compared to control cultures. Significant differences were observed only for HKDC1, ENO2, and GFAP (Figure 4).

## 4. Discussion

The principal novelty of this study is the demonstration, for the first time, of widespread glycolytic-enzyme dysregulation in the peripheral blood of AUD patients without advanced liver disease and patients with AAC, together with evidence of impaired astrocyte function, thereby identifying a potential early, non-invasive marker of alcohol-induced multisystemic stress. The bioinformatics studies and literature review carried out in this work have allowed a detailed characterization of the genes that code for 7 out of the 10 enzymes of the glycolytic pathway, highlighting their structural diversity and their involvement in metabolism and diseases (Supplementary Table S2). The identification of multiple isoforms underlines their role in different physiological and pathological contexts, from participation in adaptation to different metabolic requirements to their involvement in tumor processes, diabetes, hematological, cardiovascular, and liver diseases, as well as in mental illness and conditions related to alcohol use [39]. Additionally, these glycolytic genes can give rise to multiple transcripts which vary in length, especially in the region corresponding to the coding sequence, probably altering protein function and/or subcellular location. In some cases, such as the PFKM isoform gene, around 40 different transcripts can be generated, while for other genes, for example, the PGK1/2 and ALDA isoforms, only a single transcript was identified (Supplementary Table S2). Regarding the PGK2 isoform, it is a retrogene that is expressed only during spermatogenesis, as a result of both transcriptional and translational control [40]. Analysis of its sequence revealed that this enzyme has an authentic PGK domain (Supplementary Table S3), very similar to that of PGK1. This is in agreement with the lack of PGK activity in sperm from *Pgk2*^−/−^ male mice [40]. Non-catalytic domains have been identified in the structures of the enzymes ENO4 and ENO5. In ENO4, a dimerization/docking (D/D) domain was identified, known to have a function associated with protein–protein interaction. For ENO5, a capping domain was identified, related to functions such as regulation of enzymatic activity, structural stability, anchoring of cellular structures, and protein–protein interaction, which, to the best of our knowledge, has not been reported until now (Supplementary Tables S2 and S3). Both isoforms have high expression in some types of cancer, but their role in the pathogenesis is as yet unclear.

Regarding expression analysis in PB samples of patients with AUD, our findings provide new evidence that chronic and excessive alcohol consumption is associated with broad metabolic reprogramming, even in the absence of advanced liver disease. We note that similar glycolytic re-wiring, particularly HKDC1 up-regulation, has been reported in liver multi-omics studies of alcoholic hepatitis [8], suggesting a systemic mechanism that spans blood and target organs. Previous studies have reported GEs dysregulation in liver tissue from patients with severe alcohol-related liver diseases, including AH and AC [8]. Consistent with our findings, other studies have shown that ethanol exposure induces upregulation of glycolytic pathway genes, notably PKM2, further supporting the concept of alcohol-induced metabolic reprogramming [41]. Similarly, chronic alcohol exposure in a non-human primate model was associated with increased hepatic expression of key GEs, including PFK and ALD [42]. However, to our knowledge, this is the first study to demonstrate such alterations at the gene expression level in PB from patients with AUD without significant liver pathology (i.e., no cirrhosis or AH). We observed marked overexpression of multiple GE isoforms in PB samples from patients with AUD, including several isoforms of HK (HK1, HK2, HK3, HK4, and HKDC1), PFK (PFKP), and PK (PKM1/M2), together with a generalized overexpression of GAPDH and PGK (Figure 2, Supplementary Figure S1). Taken together, HK2, HKDC1, PKM2, ALDA, ALDC, GAPDH, and PGK form a peripheral “glycolytic signature” that could serve as a sensitive indicator of heavy drinking; its specificity versus other inflammatory states remains to be established in larger cohorts.

The application of Bonferroni correction, both globally (BfG) and by gene family (BfF), reinforces the robustness of our findings despite the exploratory nature of this study. Several key genes, including HK1, HK4, HKDC1, PFKP, ALDA, ALDC, and GAPDHS, remained significant even after stringent global correction, highlighting their strong association with the observed metabolic alterations. Other genes, such as HK2, GAPDH, and PGK2, retained significance only under family-wise adjustment (Supplementary Table S5), suggesting that their effects may be more subtle or context-specific but still biologically meaningful. The differential significance at the global versus family level may reflect distinct regulatory mechanisms, with some genes exerting broader effects and others contributing to specialized responses within their functional families. Overall, these results indicate that the observed gene expression changes provide a solid foundation for future mechanistic studies and validation in larger cohorts.

Additionally, these findings support the potential of glycolytic gene expression changes in PB as potential biomarkers of heavy alcohol use and its systemic metabolic effects, even in the absence of advanced liver damage. Notably, many of these genes are also dysregulated in liver tissue from patients with alcohol-related liver disease [8], suggesting a common mechanism of alcohol-related glycolytic dysfunction. Thus, the PB signature may reflect alcohol-induced metabolic stress that precedes or occurs independently of overt hepatic pathology.

These findings have potential application to find biomarkers of ethanol consumption and tissue damage. In this regard, ENO activity in blood has previously been explored as a biomarker of alcoholic liver disease [9]. Our study extends this concept by demonstrating the overexpression of the ALDA and ALDC isoforms in the PB of patients with AUD. ALDA, an embryonic isoform, is known to be regulated by stress signals and has been associated with increased cellular proliferative activity [43], while ALDC, predominantly expressed in neuronal tissues, is upregulated in liver tissue from AH [8]. Importantly, the expression data of both ALDA and ALDC remained significant after BfG in patients with AAC and AUD (Supplementary Tables S5 and S6). This highlights their potential central role in the metabolic alterations associated with alcohol use disorders. These findings support the potential of such a signature to serve as a sensitive, non-invasive biomarker for heavy alcohol use and its systemic effects. Furthermore, identifying these molecular changes before the onset of advanced organ damage may offer new opportunities for early diagnosis, monitoring of AUD-related pathology, and identification of high-risk patients for early intervention.

Elevated GE expression in the AUD group could reflect an alcohol-driven immune and inflammatory activation state. Chronic alcohol consumption is known to induce a persistent pro-inflammatory activation of the immune system [44], and immune cells often respond to inflammatory stimuli by shifting their metabolism toward aerobic glycolysis (a Warburg-like effect) [45]. Upregulation of glycolysis can support immune cell survival and effector functions [45], but it also engages certain GEs in moonlighting roles beyond metabolism [45,46]. For example, the inactive dimeric form of PKM2 (iPKM2) can translocate to the nucleus and co-activate HIF-1α, inducing pro-inflammatory genes such as IL-1β [47]. Noteworthy, recent results obtained in our laboratory showed a predominantly proinflammatory profile of PB monocytes in patients with AUD, characterized by elevated mRNA levels of IL-1β [37]. Isoforms such as HK2 can contribute to amplifying inflammation by promoting inflammasome activation. Moreover, during cellular activation, glycolytic intermediates and related metabolites can accumulate, modifying proteins post-translationally or acting as epigenetic mediators. Some directly alter histones, enhancing pro-inflammatory cytokine expression [45]. All these data support the notion that alcohol-induced glycolytic reprogramming in immune cells contributes to a pro-inflammatory phenotype.

Moreover, chronic ethanol metabolism generates ROS and alters redox-sensitive signaling pathways, such as those mediated by NF-κB and Mitogen-activated protein kinase MAPK, leading to dysregulated transcription of target genes [48]. This oxidative imbalance may interfere with the expression of glycolytic genes and potentially compromise the fidelity of tissue-specific gene expression programs, thereby contributing to aberrant expression in immune cells. Relevant in the context of our study is that certain glycolytic genes, such as that of HK2, have been identified as direct transcriptional targets of NF-κB [49].

Our studies also revealed that patients with AUD have aberrant overexpression of GAPDHS and PGK2 (Figure 2). Although most studies on GAPDHS in humans focus on its role in infertility, emerging evidence suggests that this isoform also functions in carcinogenesis as a metabolic switch [50]. In the case of PGK2, its overexpression has been reported in tumor tissues of patients with diverse types of adenocarcinomas, including hepatic, and is associated with a worse prognosis [51]. The ectopic activation of the testis-specific PGK2 and GAPDHS genes in circulating blood cells may signify that chronic consumption could interfere with some tissue-specific regulatory expression mechanisms, as has been documented for some genes of GEs and other metabolic enzymes [8]. This may be related to the fact that oxidative stress can influence DNA methylation and aberrant expression of metabolic genes [52], including those of some GEs [17]. Similar to the ALD isoforms, expression data of GAPDHS remained significant after BfG in patients with both AAC and AUD, underscoring its potential key role in the metabolic alterations associated with alcohol consumption. The overexpression of some non-canonical isoforms, and their association with tumoral development, poor prognosis, and drug resistance (HK2/HK3/HKDC1/ALDA/GAPDHS/PGK2) (Supplementary Table S2) in AUD patients seems relevant, especially knowing that alcohol is involved in the development of various types of cancer, and the aberrant regulation of some hexokinases can contribute to the development and progression of pathologies [53]. This finding is particularly relevant, not only because it represents the first report of simultaneous overexpression of non-canonical glycolytic isoforms in the context of AUD, but also because, given the well-established link between alcohol and the development of multiple cancer types, their aberrant regulation may contribute to tumor initiation and progression, underscoring their potential utility as markers for early cancer detection and prevention in these patients. In agreement with this notion is the result of a miRNA analysis of isolated CD14^+^ circulating monocytes of patients with AUD, carried out by us, revealing a distinctive miRNA profile that is potentially associated with tumoral development, including liver carcinogenesis, and alcoholic liver disease (ALD) through inflammation and oxidative stress [37].

Genes of GEs that showed significant differential expression in patients with AUD compared to healthy controls were subsequently analyzed in samples from individuals with AAC (Figure 3, Supplementary Figure S2). These findings suggest that expression of both canonical and non-canonical GEs (such as HK2/HKDC1/ALDOA/GAPDHS) may be rapidly modulated by acute alcohol exposure. This implies that cellular energy metabolism responds rapidly to ethanol and that a coordinated, possibly transcription factor–mediated, regulation occurs. If these alterations are repeated or sustained over time, they could contribute to ethanol-related inflammation. Our results confirm that AAC induces a rapid dysregulation of GEs, a response that may underlie the metabolic distress associated with alcohol intoxication and subsequent hangover symptoms. This early glycolytic shift broadens our understanding of alcohol-induced metabolic perturbations [54,55] and highlights how a single episode of binge drinking can perturb cellular energy homeostasis and trigger inflammatory cascades, reinforcing the potential damage linked to binge drinking. Our data support this position by showing that even an episode of acute alcohol intake can trigger gene-expression changes linked to alcohol-associated diseases.

Consistent with this, after BfG correction, the expression of ALDA, ALDC, and GAPDHS remained significant in both AAC and AUD patients (Supplementary Tables S5 and S6), suggesting their potential relevance as part of a preliminary transcriptional signature underlying the metabolic alterations associated with alcohol consumption. Cliff’s δ analysis indicated that many glycolytic genes showing significant alterations also had values approaching 1, suggesting a strong separation between groups. Notably, this pattern was observed in both AUD and AAC patients (Supplementary Tables S5 and S6). These high effect sizes support the robustness of the observed transcriptional changes and may point to their potential relevance as biomarkers of alcohol-related metabolic alterations, although further studies are needed to confirm their functional and clinical significance.

A key insight of our study is the parallel dysregulation of glycolytic genes in ethanol-exposed astrocytes, which mirrors the PB findings and underscores the translational relevance of our work. In primary mouse astrocyte cultures, we found that acute exposure to ethanol significantly increased the expression of HKDC1 and ENO2 (neuron-specific enolase, NSE), as well as a notable decrease in the astrocytic structural protein gene GFAP (Figure 2). The overexpression of HKDC1 in astrocytes is particularly interesting. HKDC1 expression is known to rise under cellular stress conditions as part of the integrated stress response [56]. In contrast, HKDC1 levels are reported to decline in aging and Alzheimer’s disease models, where its loss is associated with impaired glucose metabolism, mitochondrial dysfunction, and heightened neuroinflammation [57]. The fact that ethanol upregulates HKDC1 in astrocytes (even as aging and neurodegeneration correlate with HKDC1 loss) may suggest that alcohol triggers a stress-compensatory response in astrocytic glycolysis. Similarly, the increase in astrocytic ENO2 expression we observed could signify an early sign of neuronal stress in the culture. ENO2 (neuron-specific enolase) is typically considered a neuron-enriched GE, and abnormal levels of ENO2 have been used as a marker of neuronal injury and NDDs progression [58]. The parallel upregulation of stress-associated glycolytic genes in both PB and astrocytes reinforces the idea that blood-based metabolic biomarkers in AUD can reflect CNS cellular stress.

Ethanol effects on astrocyte gene expression extended beyond metabolic enzymes to genes related to astrocytic function. We observed a significant reduction in GFAP expression in ethanol-treated astrocytes, consistent with in vivo studies showing that chronic ethanol exposure disrupts astrocyte cytoskeletal integrity and reduces both the transcription and stability of GFAP mRNA [59]. A loss or downregulation of GFAP suggests that astrocytes may become morphologically and functionally compromised under alcohol stress, potentially affecting their ability to maintain neuronal support and blood–brain barrier integrity. We also noted downward trends (though non-significant) in the expression of the slc1a3 gene (EAAT1/GLAST, a glutamate transporter) and Park7 gene (DJ-1, an antioxidant and mitochondrial protective protein) in the presence of ethanol. Although these results should be confirmed, it is of note that even subtle decreases in EAAT1 could impair astrocyte capacity to clear extracellular glutamate, risking excitotoxicity, while lower DJ-1 suggests weakened defenses against oxidative stress. DJ-1 plays a critical role in maintaining redox balance in astrocytes and facilitating glutamate uptake; loss of DJ-1 is implicated in neurodegenerative conditions due to impaired astrocytic support functions. Therefore, the changes we observed hint that chronic alcohol exposure may initiate astrocytic dysfunction on multiple fronts, including energy metabolism, antioxidant capacity, and neurotransmitter recycling, which over time could synergistically reduce neuronal resilience.

The interplay between GE dysregulation and oxidative stress thus emerges as a potential mechanism for alcohol-related astrocyte injury that may contribute to neurodegeneration. On one hand, altered glycolytic flux in astrocytes can disturb redox homeostasis. PKM2, which we found elevated in PB of patients with AUD, is a central regulator of glycolytic flux; its activity level dictates how much glucose is channeled into oxidative phosphorylation versus diverted into the pentose-phosphate pathway for NADPH production [60]. A less active PKM2 (commonly seen in stress conditions) can divert metabolites toward NADPH generation, bolstering antioxidant defense. Conversely, dysregulated or overactive PKM2 might limit this flexibility, leading to insufficient NADPH and thus reduced glutathione to neutralize ROS. On the other hand, HK2, which was upregulated in AUD patients, has been directly linked to autophagy regulation. Uniquely among hexokinase isoforms, HK2 can bind and sequester mTORC1 on the lysosomal surface, thereby activating autophagy under glucose-starvation or stress conditions [61]. If chronic alcohol misuse leads to aberrant HK2 expression or localization, it could disrupt normal autophagy signaling. Indeed, alcohol-induced oxidative stress is known to impair autophagy and mitophagy in the brain, resulting in the accumulation of damaged proteins and organelles [62]. Impaired autophagy in astrocytes would exacerbate oxidative injury, as cells lose the capacity to clear dysfunctional mitochondria (a major source of ROS) and other toxic substrates. Thus, the alcohol-driven GEs transcript changes we identified (e.g., high HK2 and PKM2) may contribute to a vicious cycle of oxidative stress and autophagy dysfunction in astrocytes. Over time, this could set the stage for neurodegenerative processes: damaged, energetically compromised astrocytes are less able to support neurons, control glutamate levels, or detoxify ROS, thereby increasing neuronal vulnerability. Importantly, similar disturbances in GEs, particularly HK2, were also observed after AAC, suggesting that binge-drinking episodes can act as early triggers that promote inflammation, redox imbalance, and metabolic stress, which may help explain their impact on organs such as the liver and the central nervous system [63,64].

In this paper, we presented the results of our exploratory study and, therefore, have to acknowledge the relatively small sample size and cross-sectional design, as well as the lack of experiments specifically addressing mechanistic pathways or measuring antioxidant activity. In particular, we did not determine baseline ROS levels relative to treated groups or perform antioxidant activity assays, which would have strengthened the interpretation of our findings. These issues will be addressed in our follow-up studies.

## 5. Conclusions

Our results provide evidence of dysregulated GE expression in PB samples of patients with both chronic and acute excessive alcohol intake as well as in vitro cultures of ethanol-exposed mouse astrocytes. In AUD patients and individuals with AAC, significant overexpression of several GEs was observed, including isoforms of the regulatory enzymes HK, PFK, and PK. Furthermore, overexpression of several ALD, GADPH, and PGK isoforms was detected. Simultaneous overexpression of non-canonical glycolytic isoforms, such as HK2, a well-known marker of cancer metabolism, also showed differential expression. Furthermore, several isoforms not typically associated with the canonical glycolytic pathway were also differentially expressed, further highlighting the complexity of metabolic reprogramming in patients with AUD. Overall, our findings highlight the potential utility of dysregulated GE expression as a non-invasive biomarker of alcohol-induced metabolic stress and opens the possibility of targeting specific glycolytic isoforms (e.g., HK2 or PKM2) as therapeutic strategies for modulating the metabolism–inflammation axis in alcohol-related diseases beyond liver pathology. While limitations such as small sample size and cross-sectional design constrain the mechanistic depth of our conclusions, these findings lay the groundwork for future studies exploring glycolytic modulation support as a strategy to preserve astrocytic and neuronal function under chronic alcohol exposure.

## Figures and Tables

**Table 1:** Demographic and clinical characteristics of AUD patients and healthy controls.

Variable	AUD Patients (*n* = 11)	Controls (*n* = 7)	*p*-Value
Age (years)	54.51 (10.43)	52.85 (4.38)	0.64
Male (n*)/Female (n*)	9 (81.80)/3(27.27)	4 (57.14)/3 (42.85)	0.52
Total bilirubin (mg/dL)	0.80 (0.51)	0.82 (0.29)	0.84
AST (U/L)	117 (98.65)	17.88 (4.94)	0.049
ALT (U/L)	64.27 (43.43)	27.86 (18)	0.034
GGT (U/L)	348.29 (518)	21.14 (11.23)	0.17
ALP (U/L)	91.45 (29.22)	55.86 (9.55)	0.003
Proteins (g/dL)	7.3 (0.70)	7.50 (0.49)	0.89
Albumin (g/dL)	4.5 (0.32)	4.77 (0.16)	0.11
Ferritin (ng/mL)	205 (112.62)	98.96 (91.62)	0.15
Hemoglobin (g/dL)	16.10 (1.46)	15.11 (1.34)	0.19
Leukocytes (×10^3^ cells/μL)	8.77 (3.41)	6.20 (8.29)	0.045
Neutrophils (×10^3^ cells/μL)	5.73 (2.77)	3.67 (6.16)	0.043
Platelets (×10^3^ cells/μL)	252.27 (80.83)	233.42 (66.40)	0.62
Total cholesterol (mg/dL)	175 (42.43)	197 (34.38)	0.29
Triglycerides (mg/dL)	106.2 (55.13)	90.06 (46.07)	0.55

Quantitative variables are presented as mean (standard deviation), and qualitative variables as * number of cases (percentage (%)). Aspartate aminotransferase (AST), alanine aminotransferase (ALT), gamma-glutamyl transferase (GGT), and alkaline phosphatase (ALP). *p* < 0.05.

**Table 2:** Demographic and clinical characteristics of acute alcohol consumption (AAC) patients and healthy controls.

Variable	AAC Patients (*n* = 7)	Controls (*n* = 7)	*p*-Value
Age (years)	18.71 (3.37)	22.57 (2.37)	0.043
Male (n*)/Female (n*)	6 (85.71)/1 (14.18)	4 (57.14)/3 (42.85)	0.272
AST (U/L)	33.29 (13.64)	18.83 (4.60)	0.040
ALT (U/L)	18.29 (4.46)	23.33 (18.25)	0.572
ALP (U/L)	105.01 (54.96)	60.17 (13.43)	0.094
GGT (U/L)	18.71 (5.09)	15.67 (8.81)	0.513
LDH	228.71 (96.88)	159.67 (11.79)	0.134
Leukocytes (×10^3^ cells/μL)	8.19 (1.85)	5.18 (1.35)	0.010

Quantitative variables are presented as mean (standard deviation), and qualitative variables as * number of cases (percentage (%)). Aspartate aminotransferase (AST), alanine aminotransferase (ALT), alkaline phosphatase (ALP), gamma-glutamyl transferase (GGT), and lactate dehydrogenase (LDH). *p* < 0.05.

## Data Availability

The data presented in this study are available upon request from the corresponding author. The data are not publicly available for ethical reasons.

## Supplementary Materials

The following supporting information can be downloaded at: https://www.mdpi.com/article/10.3390/antiox14091143/s1, Figure S1. Heatmap of glycolytic gene expression in patients with AUD. Normalized expression levels were log_2_-transformed (log_2_[X + 1]) and visualized using GraphPad Prism; Figure S2: Heatmap of glycolytic gene expression in patients with AAC. Normalized expression levels were log_2_-transformed (log_2_[X + 1]) and visualized using GraphPad Prism; Table S1: Nucleotide sequences of primer pairs applied for the RT-PCR; Table S2: Molecular and cellular features of some glycolytic enzymes [8,11,53,58,65,66,67,68,,69,70,71,72,73,74,75,76,77,78,79,80,81,,82,83,84,85,86,87,88,89,90,91,92,93,94,95,96,97,98,,99,100,101,102,103,104,105,106,107,,108,109,110,111,112,113,114,115,116,117]; Table S3: E-values obtained for sequence similarity analysis of certain regions of glycolytic enzymes described in this study with known canonical protein domains, using the servers and software specified below. Table S4: Effect size analysis using Cliff’s delta (δ) for patients with AUD and ACC; Table S5: Raw and Bonferroni-adjusted *p*-values for glycolytic gene expression in peripheral blood of patients with AUD; Table S6: Raw and Bonferroni-adjusted *p*-values for glycolytic gene expression in peripheral blood of patients with AAC.
