# Supplementary material for: Dysregulated Expression of Canonical and Non-Canonical Glycolytic Enzyme Isoforms in Peripheral Blood from Subjects with Alcohol Use Disorder and from Individuals with Acute Alcohol Consumption
Source: Antioxidants (Basel). 2025 Sep 22;14(9):1143. doi: 10.3390/antiox14091143 (PMC12466832; doi:10.3390/antiox14091143)
Supplement: Supplementary file 1 [file antioxidants-14-01143-s001.zip › antioxidants-3832208-supplementary.pdf]

Supplementary Material

Supplementary Table S1. Nucleotide sequences of primer pairs applied for the RT-PCR

Gene	Primers
**Human**	
**Hexokinase**	
HK1	FW: 5´-TCCTCGTCAAGACAGTGTGC-3´<br>RV: 5´-TGGAGAAGTGTGGATGAAGC-3´
HK2	FW: 5´-GTCTCCACTGAGGATGTGAG-3´<br>RV: 5´-GGCAAATGCTTGTAGATGAG-3´
HK3	FW: 5´-AAGAGGTGATGCTGGGTGCTG-3´<br>RV: 5´-AATGAGGGTGCTCCTGTCCAA-3´
HK4	FW: 5´-AGGAGGACCTGAAGAAGGTGAT-3´<br>RV: 5´-TCGAAGAGCATCTCAGCAGTG-3´
HKDC1	FW: 5´-TATGACGACCCCTACTGCGA-3´<br>RV: 5´-ACTCAGTGCGAATGTCCTCC-3´
**Phosphofructokinase**	
PFKM	FW: 5´-TATCTAAGAGTGGTTCGCAC-3´<br>RV: 5´-ACTGATCTGTTCAAAGCTCT-3´
PFKL	FW: 5´-CGATAACGACTTCTGCGGCA-3´<br>RV: 5´-CCCAGCCTCTCACACATGAA-3´
PFKP	FW: 5´-GGGCAACCTGAACACCTACA-3´<br>RV: 5´-GCATCTGTGTGGCGATCTCT-3´
**Aldolase**	
ALDA	FW: 5´-GCCCGTTATGCCAGTATCTG-3´<br>RV: 5´-TGTAGACAGCAGCCAGCACC-3´
ALDB	FW: 5´-AGCCTCGCTATCCAGGAAAACG-3´<br>RV: 5´-CTTGTAGACAGCAGCCAGGACC-3´
ALDC*	FW: 5´-GCCATGCCTGTCCCATCAAG-3´<br>RV: 5´-GCACGCCCATAGGAGAAGGT-3´
**Glyceraldehyde-3-phosphate dehydrogenase**	
GADPH	FW: 5´-CCATGAGAAGTATGACAACAGCC-3´<br>RV: 5´-GGGTGCTAAGCAGTTGGTG-3´
GADPH-S	FW: 5´-CATGGAGAAGGGTGTTAAGG-3´<br>RV: 5´-GGGATCTGTTTGGGCTCTT-3´
PGK1	FW: 5´-ACTGTTTCACTACTCAGCATGGA-3´<br>RV: 5´-TCAGCAATATAGACATCTGATCC-3´
PGK2	FW: 5´-TGACGAGAACGCTCAGGTTG-3´<br>RV: 5´-GCCCATTCCAAACAATTAGC-3´
**Enolase**	
ENO1	FW: 5´-TTCGGTCACCTGTTGGCTAC-3´<br>RV: 5´-GGTGGAAAGTGAGGCGAGAA-3´
ENO2*	FW: 5´-GGTGAAGGAAGCCATCGACA-3´<br>RV: 5´-GTCCCCAGTGATGTATCGGG-3´
ENO3	FW: 5´-CTGTCTGCTGCTGAAGGTCA-3´<br>RV: 5´-TTCGGGTTACGGAACTTG-3´
**Pyruvate kinase**	
PK_M1/2	FW: 5´-TGTTTGCGTCATTCATCCG-3´<br>RV: 5´-TAGATCACCACGAGCCAC-3´
PK_L/R	FW: 5´-TGGGGTGGACATCGTCTTTG-3´

	RV: 5´-TGATGATCTTGATGCCGTGT-3´
18S ribosomal RNA (18S rRNA)	FW: 5´-GGATGAGGTGGAACGTGTGA-3´<br>RV:5´-CCAGACCATTGGCTAGGACC-3´
**Mouse**	
HK-2_m	FW: 5´-TTCCGTGTGCTCCGAGTAAG-3´<br>RV: -ACGCCACTGGACTTGAACC-3´
GAPDH_m	FW: 5´-TGAAGCAGGCATCTGAGGG-3´<br>RV: -CGAAGGTGGAAGAGTGGGA-3´
HKDC1_m	FW:5´-ACTGTGATGTCCGCTTCCTC-3´<br>RV: -CGTACTCTAGTTCAGCCCGC-3´
Glial fibrillary acidic protein (GFAP)	FW:5´-CTGATGTCTACCAGGCGGAG-3´<br>RV: 5´-TCCAAATCCACACGAGCCAG-3´
Glial high affinity glutamate transporter (EAAT2)	FW:5´-AGTCAATGTGGTGGGCGATT-3´<br>RV: 5´-TCGTCGTAAATGGACTGCGT-3´
Parkinsonism associated deglycase (PARK7 or DJ-1)	FW: 5´-CTGTTGGCTCACGAAGTAGG-3´<br>RV: 5´-GGCTTGGGCTCTAGTCTTTG-3´
TATA-box binding protein (TBP)	FW: 5´-GAAGCTGCGGTACAATTCCAG-3´<br>RV: 5´-CCCCTTGTACCCTTCACCAAT-3´

Legend
*** Human- and mouse-specific oligonucleotides**

Supplementary Table S2. Molecular and cellular features of some glycolytic enzymes

Gene	GENE ID	Genomic location	mRNA transcript variants (Accession Number)[1]		Associated domains	Tissue/cell type expression	Subcellular location	Clinical relevance	Reference
			Long	Small					
**Hexokinase**									
Hexokinase 1 (HK1)	3098	10	NM_001358263.1<br>NM_001322364.2<br>NM_001358263.1<br>NM_033497.3<br>NM_033498.3<br>NM_001322365.2<br>NM_033500.2<br>XM_024447969.2<br>XM_047425136.1<br>XM_047425137.1	NM_000188.3<br>NM_033496.3<br>NM_001322366.1<br>NM_000188.3<br>NM_001322367.1<br>XM_047425139.1	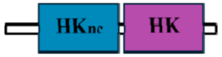	Embryonic and human fetal tissues (HFT)<br>Adult tissue (Ubiquitous)	Cytosol*♦<br>Nucl*<br>Mitoch*♦	Cancer<br>Diabetes<br>Diseases<br>Neurodevelopment<br>Inflammatory Diseases<br>Prognostic marker in cancer	[1–5]
Hexokinase 2 (HK2)	3099	2	NM_000189.5<br>XM_005264280.3<br>XM_011532807.3<br>XM_017003945.3<br>XM_047444084.1<br>NM_001371525.1<br>XM_047444085.1		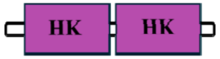	Embryonic development<br>Insulin-sensitive tissues<br>Diverse tumoral tissue	Cytosol*♦<br>Nucl*♦<br>Mitoch*♦	Cancer<br>Inflammatory diseases<br>Neurodegenerative disease (NDD)<br>Alcoholic liver diseases<br>Considered a hallmark of cancer cells<br>Prognostic marker cancer<br>High mRNA expression positively correlated with a progression of cirrhosis to HCC and a low survival rate	[1–3,5–9]
Hexokinase 3 (HK3)	3101	5	NM_002115.3<br>XM_047417134.1<br>XM_011534540.3		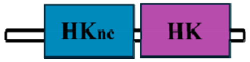	Low expression in adult<br>Enriched in lymphoid tissue and bone marrow<br>Hematopoietic cells and	Cytosol*♦<br>N. periphery♦<br>MAMs♦<br>Mitoch*	Cancer<br>Sepsis-induced acute lung injury (ALI)<br>Prognostic marker in cancer	[1–3,5,10–12]

						subsets of immune cells			
Hexokinase 4 (HK4)/ Glucokinase (GCK)	2645	7	NM_000162.5<br>NM_001354800.1	NM_001354801.1<br>NM_001354802.1<br>XM_024446707.2<br>NM_033507.3<br>NM_033508.3<br>NM_001354803.2	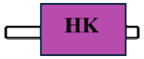	Expression in all tissues at variable levels<br>High expression in brain, liver, pituitary gland, heart muscle and Immune cells	Cytosol♦<br>Nucleus*♦<br>Mitoch*♦	Cancer<br>Insulin sensitivity<br>Diabetes<br><br>Prognostic marker in cancer<br><br>Associated with lipogenesis and fatty liver	[1–3,5,13–16]
Hexokinase domain containing 1 (HKDC1)	80201	10	NM_025130.4<br>XM_011540195.3<br>XR_007061989.1	XM_047425784.1	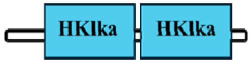	Expression in several tissues<br>Enriched in brain, intestine, kidney retina, placenta and immune cell	Cytosol*<br>Nucl*<br>Mitoch*♦<br>Lysos*♦	Cancer<br>Liver diseases<br>Alcoholic hepatitis<br>Diabetes<br>Prognostic marker in cancer<br>Biomarker associated with the decreased survival in AH<br>Biomarker and therapeutic target for AH	[1–3,5,17–19]
**Phosphofructokinase**									
Phosphofructokinase, platelet (PFKP)	5214	10	NM_002627.5<br>XM_005252466.5<br>NM_001323071.2<br>NM_001410880.1<br>NM_002627.5<br>NM_001323068.2<br>NM_001323069.2<br>NM_001323067.2<br>XM_047425350.1<br>NM_001323072.2<br>NM_001242339.2<br>XM_006717449.2<br>NM_001345944.1	NM_001323074.1<br>NM_001323070.1<br>NM_001323073.1	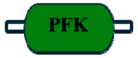	Expression in several tissues<br>Enriched in brain, retina, platelet and immune cells	Cytosol*♦<br>Nucl*♦	Cancer<br>Cardiac disease<br>Liver fibrosis<br>Prognostic marker in cancer	[1–3,20–24]
Phosphofructokinase, muscle (PFKM)	5213	12	NM_000289.6<br>NM_001354741.2<br>NM_001354742.2<br>NM_001354747.2<br>XM_047428999.1<br>XM_011538487.2	NM_001354740.1<br>XM_047429003.1<br>NM_001354744.2<br>NM_001354745.2<br>NR_148957.2<br>NR_148954.2	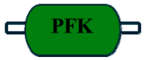	Expression in several tissues<br>Enriched in muscle cells, muller glia cells, tongue	Cytosol*♦<br>Nucl*<br>E. reticulum*♦<br>Cytosk*♦	Cancer<br>Type VII glycogen storage disease (GSDVII)<br>Cardiac and hematological	[1–3,24–26]

			XM_047428999.1<br>XM_047429000.1<br>NM_001354743.2<br>NR_148955.1<br>XM_005268976.4<br>NM_001166686.2<br>XM_047429001.1<br>XM_024449020.2<br>XM_047429002.1<br>XM_024449021.2<br>XM_047429004.1<br>NM_001354736.1<br>NM_001354737.1<br>XM_017019469.2<br>NM_001354735.1<br>NM_001354739.1<br>XM_005268974.2<br>NM_001354738.1	NR_148958.2<br>NM_000289.6<br>NM_001354746.2<br>NM_001363619.2<br>NR_148956.2<br>NR_148959.2<br>NM_001354748.2<br>XM_024449022.2<br>NM_001166688.2<br>NM_001166687.2		and testis - Spermatocytes<br><br>Low expression in blood and immune cells		disorder<br>Predictor of survival time, recurrence, and risk to some type of cancer<br>High mRNA expression positively correlated with a progression of cirrhosis to HCC and low survival	
Phosphofructokinase, liver type (PFKL)	5211	21	NM_002626.6<br>XM_047440822.1<br>XM_024452085.2<br>XM_006724011.2<br>NM_001002021.3<br>XM_047440825.1<br>XM_017028369.2<br>XM_005261135.4<br>NM_002626.6	XM_047440824.1<br>XM_047440823.1<br>XM_005261137.5<br>XM_011529603.3	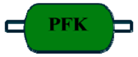	Expression in several tissues<br>Enriched in intestine, liver, kidney, testicle, placenta and immune cells	Cytosol *♦<br>Nucl*<br>Mitoch*	Cancer<br>Hepatic fibrosis<br>Cognitive disabilities<br>Biomarker for cancer prognosis and therapeutic target<br>High mRNA expression positively correlated with a progression of cirrhosis to HCC and low survival rate	[1–3,24,27,28]
**Aldolase**									
Aldolase A (ALDA)	226	16	NM_184043.2<br>NM_001127617.2	NM_001243177.4<br>NM_184041.5	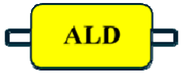	Embryonic and fetal tissues<br><br>After birth is constitutively expressed in most adult tissues	Cytosol*♦<br>Nucleus*♦<br>Cytosk*♦<br>Lysosome*<br>Mitoch*	Cancer<br>Hematological disorder<br>Diagnosis and prediction in patients with leukaemia<br>High mRNA expression in cirrhosis correlates with the risk of	[1–3,29,30]

								developing liver cancer	
Aldolase B (ALDB)	229	9	NM_000035.4		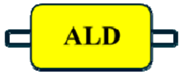	Embryonic development In adult tissue, mainly expressed in renal tubules, hepatocytes, small intestine and duodenum.	Cytosol*♦ Nucl*♦ Mitoch*	Cancer<br>Liver dysfunction<br>Biomarker for cancer prognosis<br>Modulator of the response to immunotherapy in liver cancer<br>Proteins associated with future diabetes risk	[1–3,31,32]
Aldolase C (ALDC)	230	17	NM_005165.3<br>XM_011524556.3<br>XM_005257949.3		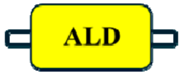	Expressed in in most tissues Enriched in the brain, retina and immune cells	Cytosol*♦ Nucl* Vesicles*	Cancer<br>NDD<br>Mental illness<br>Brain injuries<br>Biomarker for cancer prognosis and therapeutic target<br>Biomarker of astroglial injury	[1–3,33,34]
**Glyceraldehyde-3-phosphate dehydrogenase**									
Glyceraldehyde-3-phosphate dehydrogenase (GADPH)	2597	12	NM_002046.7<br>NM_001289745.3<br>NM_002046.7<br>NM_001357943.2<br>NM_001256799.3		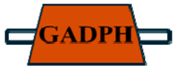	Ubiquitous expression	Cytosol*♦ Nucl*♦ P. membr*♦	Cancer<br>NDD<br>Potential therapeutic target for cancer and neurodegenerative disorders	[1–3,35–37]
Glyceraldehyde-3-phosphate dehydrogenase, sperm-specific (GADPHS)	26330	19	NM_014364.5		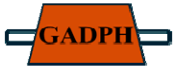	High expression in testis (spermatids and oocytes) Expression slow in thyroid gland, pancreas, skin, lymph node	Cytosol*♦ Fibrous sheath*♦	Cancer<br>NDD<br>Male infertility	[1–3,38,39]
**Phosphoglycerate kinase**									

Phosphoglycerate kinase 1 (PGK1)	5230	X	NM_000291.4		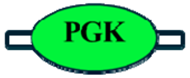	Ubiquitous expression	Cytosol*♦<br>Nucl*♦<br>Mitch*♦	Cancer<br>Glycogen storage disease<br>Hematological disorder<br>Mental illness<br>NDD<br>Prognostic marker in different types of cancer ant therapeutic target<br>Marker of resistance to chemotherapy drugs	[1–3,40–42]
Phosphoglycerate Kinase 2 (PGK2)	5232	6	NM_138733.5		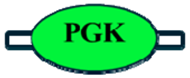	Expression of developmental stage-specific manner during spermatogenesis High expression in testis (spermatids and oocytes)	Cytosol*<br>Nucl *<br>Fibrous sheath*♦	Cancer<br>Asthenozoospermia<br>Expression in cells lines tumoral (myeloid leukemia, lymphoma, liver and lung)	[1–3,43,44]
**Enolase**									
Enolase 1 (ENO1) / α-enolase	2023	1	NM_001428.5<br>NM_001353346.3<br>NM_001201483.4		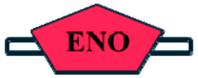	Ubiquitous expression	Cytosol*♦<br>Nucl*♦<br>P. memb*<br>Mitoch*♦<br>Cytosk♦	Cancer<br>Glycogen storage disease<br>Diabetes<br>Inflammatory diseases<br>NDD<br>Diagnostic and progression marker in different types of cancer	[1–3,45–47]
Enolase 2 (ENO2) / γ-enolase	2026	12	NM_001975.3		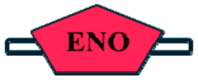	CNS peripheral nerves and pancreatic islets	Cytosol♦<br>Nucl*♦<br>P. memb*♦<br>Mitoch*♦	Cancer<br>Cardiovascular disease<br>NDD<br>Diagnostic marker in different types of cancer<br>Critical biomarker	[1–3,46,47]

								for NDD and neurological conditions	
Enolase 3 (ENO3) / β-enolase	2027	17	XM_006532161.5<br>XM_006532162.4	XM_030245527.2<br>NM_001276285.1<br>NM_007933.3<br>NM_001136062.2<br>XM_036156290.1	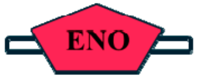	Muscle<br>Liver	Cytosol*♦<br>Nucl*<br>P. memb*♦<br>Mitoch*	Cancer<br>Muscle injuries<br>Metabolic myopathy<br>Prognostic marker in pancreatic cancer	[1–3,46,47]
Enolase 4 (ENO4)	387712	10	XM_006717835.4	NM_001242699.2<br>XM_011539797.4<br>XM_047425228.1<br>XM_005269815.4	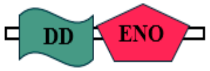	Choroid plexus, Fallopian tube Lung, testis, thyroid<br>Cells ciliated	Cytosol*<br>Vesicles*	Male infertility<br>Autoimmune thyroid diseases	[1–3,48,49]
Enolase 5 (ENO5) / Mitochondrial enolase superfamily member 1 rTSbesa (ENOSF1)	55556	18	NM_017512.7<br>XM_047437621.1<br>XM_024451208.2<br>XM_024451209.2<br>XM_047437614.1<br>NM_001354065.2<br>XM_024451210.2<br>NM_001318760.2<br>XM_047437612.1<br>XM_024451203.2<br>NM_001354066.2<br>NM_017512.7<br>XM_024451201.2<br>XM_047437613.1<br>NM_001354068.2<br>XM_024451204.2<br>XM_024451202.2<br>NM_202758.5<br>NM_001354067.2	XM_047437611.1<br>XM_047437617.1<br>XM_047437618.1<br>XM_047437620.1<br>XM_047437616.1<br>XM_047437615.1<br>XM_047437619.1<br>XM_047437622.1	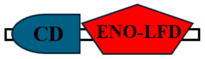	Liver<br>Tissues with gastric cancer	Cytosol*♦<br>Nucl*<br>Mitoch*♦	Overexpression in methotrexate and 5-fluorouracil resistant cell lines<br>Marker of severe toxicity in anti-cancer drugs treated cancer patients<br>Predictor of treatment response and survival in patients with cancer	[1–3,50–53]
				**Pyruvate kinase**					
Pyruvate kinase L/R (PKL/R)	5313	1	XM_047422591.1<br>XM_047422592.1	NM_000298.6<br>XM_011509640.4<br>XM_006711386.5<br>NM_181871.4<br>XM_017001493.1	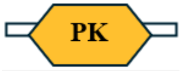	Ubiquitous expression<br>**PKL**<br>Liver and kidney<br>**PKR**<br>Blood and immune cells	Cytosol*♦<br>Nucleus*<br>Mitoch*	Cancer<br>Hematological disorder<br>Chronic liver disease<br>NAFLD<br>Predictive marker for the treatment of cancer	[1–3,54–56]

Pyruvate kinase M (PKM1/2)	5315	15	NM_002654.6<br>XM_005254445.6<br>NM_001206797.3<br>NM_002654.6<br>NM_182471.4<br>NM_001206798.3<br>NM_182470.4<br>XM_011521670.2<br>NM_001316318.2<br>NM_001411081.1<br>NM_001206796.3<br>XM_047432666.1<br>XM_047432664.1<br>XM_047432665.1<br>XM_005254443.2<br>NM_001206799.2	XM_047432662.1<br>XM_047432663.1	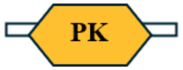	Ubiquitous expression<br>**PKM1**<br>Expressed in tissue adult and differentiated cells<br>**PKM2**<br>Tumoral cells and embryos/fetal cells<br>Immune cell	Cytosol*♦<br>Nucl*♦<br>Mitoch*	Cancer<br>Hematological disorder<br>NDD<br>Bones disease<br>Inflammation<br>Prognostic marker in diverse types of cancer	[1–3,56,57]

Genes of glycolytic enzymes (GEs) identified through genomic analyses in humans. GE sequences were retrieved from the National Center for Biotechnology Information (NCBI) database. Domain and motif identification was performed using Protein BLAST (NCBI), Conserved Domain Database (CDD), InterProScan (EMBL-EBI), and the Simple Modular Architecture Research Tool (SMART), with e-values ranging from 0.0 to 5×10^{-13}, ensuring high-confidence detection. Subcellular localization predictions were based on either experimental evidence or computational tools, including WoLF PSORT, DeepLoc, Cell-Ploc, and the Human Protein Atlas (HPA)·. Notation for subcellular localization: * predicted by bioinformatics analyses; ♦ experimentally validated. **Localization symbology**: Cytosol, Cytosol; Nucl, nucleus, Mitoch, Mitochondria; P. memb, plasma membrane; Lys, lysosome; Cytosk, cytoskeleton. N. periphery, nuclear periphery; MAMs, mitochondria-associated membranes. * In silico prediction, ♦ localization established through experimental studies. **Domain symbology**: 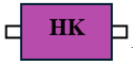

HK domain with catalytic activity, 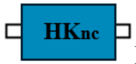

HK domain without catalytic activity; 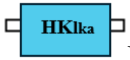

HK domain with low kinase activity, 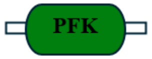

PFK domain, 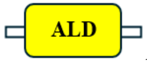

ALDO domain, 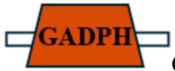

GAPDH domain, 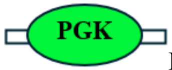

PGK domain, 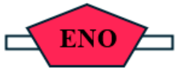

ENO domain, 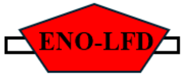

ENO-L-fuconate dehydratase (ENO-LFD) domain, 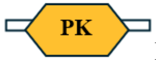

PK domain, 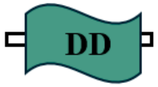

Dimerization/docking (D/D) domain, 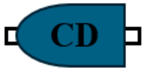

capping domain.

[1] mRNA transcript variants and their accession numbers, which identify specific sequences in the NCBI database

Supplementary Table S3. E-values obtained for sequence similarity analysis of certain regions of glycolytic enzymes described in this study with known canonical protein domains, using the servers and software specified below.

Enzyme	Gene ID	Accession number [1]	e-value									
			Domains described									
			HK	PFK	ALD	GADPH	PGK	ENO	ENO-LFD	PK	CD	D/D
HK1	3098	NM_001358263.1	2e-^{71} - 1.3e-^{91}									
HK2	3099	NM_000189.5	5.4e-^{78} - 1.5e-^{91}									
HK3	3101	NM_002115.3	6.2e-^{65} - 6.2e-^{65}									
HK4	2645	NM_000162.5	3.10e-^{92}									
HKDC1	80201	NM_025130.4	4.3e-^{71}- 1.1e-^{84}									
PFKP	5214	NM_002627.5		0.0								
PFKM	5213	NM_000289.6		0.0								
PFKL	5211	NM_002626.6		0.0								
ALDA	226	NM_001243177.4			2.2e-^{186}							
ALDB	229	NM_000035.4			6.6e-^{177}							
ALDC	230	NM_005165.3										
GADPH	2597	NM_002046.7				3.31e-^{172}						
GADPHS	26330	NM_014364.5				1.60e-^{166}						
PGK1	5230	NM_000291.4					0.0					
PGK2	5232	NM_138733.5					0.0					
ENO1	2023	NM_001428.5						8.99e-^{206}				
ENO2	2026	NM_001975.3						4.63e-^{208}				
ENO3	2027	NM_053013.4						5.8e-^{200}				
ENO4	387712	NM_001242699.2						8.07e-^{53}				4.44e-^{35}
ENOSF1	55556	NM_017512.7							2.78e-^{60}		5e-^{13}	
PKL/R	5313	NM_000298.6								0.0		
PKM1/2	5315	NM_002654.6								0.0		

Legend

E-values obtained from the sequence similarity analysis of selected regions of glycolytic enzymes with canonical protein domains (HK, PFK, ALD, GADPH, PGK, ENO, ENO-LFD, PK, CD, D/D). Domains were identified using the Conserved Domain Database (CDD), InterProScan (EMBL-EBI), and the Simple Modular Architecture Research Tool (SMART).

[1] Accession numbers correspond to the NCBI database.

Supplementary Table S4. Effect size analysis using Cliff's delta (δ) for patients with AUD and ACC

	**AUD**	**ACC**
**Gen**	**|δ|**	**|δ|**
HK1	0.94	0.63
HK2	0.81	0.90
HK3	0.71	0.51
HK4	0.89	0.67
HKDC1	0.92	0.91
PFPL	0.32	0.71
PFKP	1	0.87
PKM1M2	0.76	0.59
PKLR	0.06	
ALDA	0.95	0.71
ALDB	0.22	
ALDC	0.84	0.79
GADPH	0.71	0.38
GADPHS	1	0.79
PGK1	0.66	0.75
PGK2	0.79	0.57
ENO1	0.38	
ENO2	0.52	
ENO3	0.66	

Legend

Absolute values of Cliff's δ (|δ|) indicate the magnitude of the effect size. Effect size interpretation is as follows: |δ| = 0 indicates no difference, and |δ| = 1 indicates the maximum possible.

Supplementary Table S5. Raw and Bonferroni-adjusted p-values for glycolytic gene expression in peripheral blood of patients with AUD

Family	Gene	p_raw	Significant_global	Significant_family
HK	HK1*	0.0007	**Yes**	**Yes**
	HK2 #	0.0079	No	**Yes**
	HK3	0.0185	No	No
	HK4*	0.0021	**Yes**	**Yes**
	HKDC1*	0.0012	**Yes**	**Yes**
PFK	PFKL	0.371	No	No
	PFKP *	0.0006	**Yes**	**Yes**
PK	PKM1M2 #	0.025	No	Yes
	PMLR	0.8601	No	No
ALD	ALDA*	0.0012	**Yes**	**Yes**
	ALDB	0.669	No	No
	ALDC*	0.0023	**Yes**	**Yes**
GAPDH	GADPH #	0.0185	No	Yes
	GADPHS*	0.0002	**Yes**	**Yes**
PGK	PGK1	0.033	No	No
	PGK2 #	0.0103	No	**Yes**
ENO	ENO1	0.2949	No	No
	ENO2	0.1375	No	No
	ENO3	0.0513	Not	No

Legend

Family: functional gene family; Gene: gene symbol; p_raw: raw p-value obtained using the Mann–Whitney test; Significant_global: Significant (Yes)/Not significant (No) depending on whether p_Bonferroni_global (BG) < α; Significant_family: Significant/Not significant depending on whether p_Bonferroni_family (BfG) < α. p_Bonferroni_global: Bonferroni-adjusted p-value considering all genes together (n = 19; α = 0.05/19 ≈ 0.0026). p_Bonferroni_family: Bonferroni-adjusted p-value calculated within each gene family (α = 0.05 / number of genes in the family).

Genes marked with * are significant after Bonferroni adjustment for all genes (BfG); genes marked with # are significant after Bonferroni adjustment within their respective gene family (BfF).

Supplementary Table S6. Raw and Bonferroni-adjusted p-values for glycolytic gene expression in peripheral blood of patients with AAC

**Family**	**Gene**	**p_raw**	**Significant_global**	**Significant_family**
HK	HK1*	0.029	No	**Yes**
	HK2 #	0.0047	No	**Yes**
	HK3	0.12	No	No
	HK4 #	0.0379	No	**Yes**
	HKDC1 #	0.0047	No	**Yes**
PFK	PFKL	0.0515	No	No
	PFKP #	0.0082	No	**Yes**
PK	PKM1M2 #	0.0728	No	**Yes**
ALD	ALDA*	0.0012	**Yes**	**Yes**
	ALDC*	0.0023	**Yes**	**Yes**
GAPDH	GADPH #	0.0185	No	Yes
	GADPHS #	0.0002	**Yes**	**Yes**
PGK	PGK1	0.033	No	No
	PGK2 #	0.0103	No	**Yes**

Legend

Family: functional gene family; Gene: gene symbol; p_raw: raw p-value obtained using the Mann–Whitney test p_; Significant_global: Significant (Yes)/Not significant (No) depending on whether p_Bonferroni_global < α; Significant_family: Significant/Not significant depending on whether p_Bonferroni_family < α. p_Bonferroni_global: Bonferroni-adjusted p-value considering all genes together ($n = 14$; $\alpha = 0.05/14 \approx 0.0036$); p_Bonferroni_family: Bonferroni-adjusted p-value calculated within each gene family ($\alpha = 0.05$ / number of genes in the family).

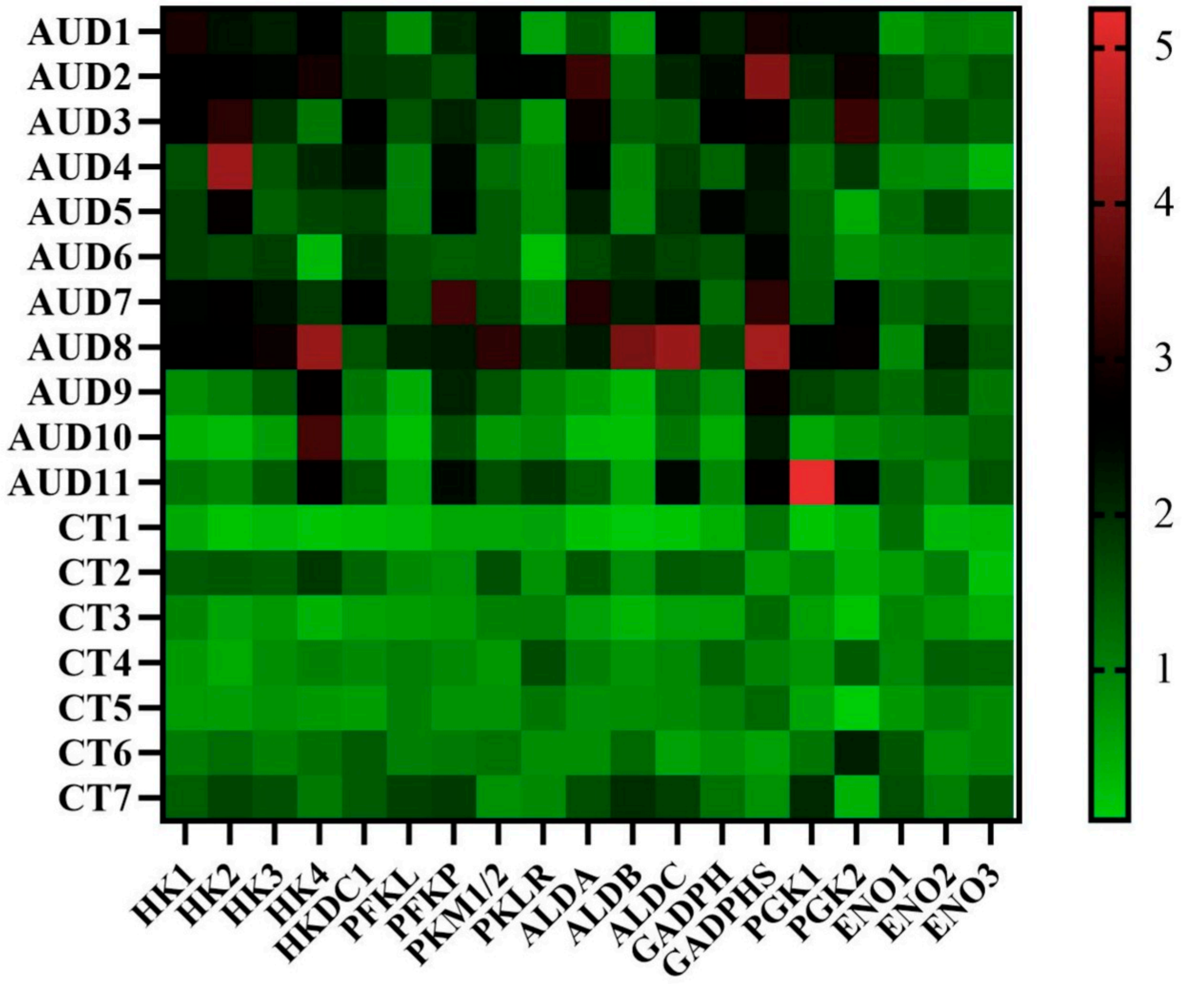

Supplementary Figure S1. Heatmap of glycolytic gene expression in patients with AUD. Normalized expression levels were log_2-transformed ($log_2[X+1]$) and visualized using GraphPad Prism.

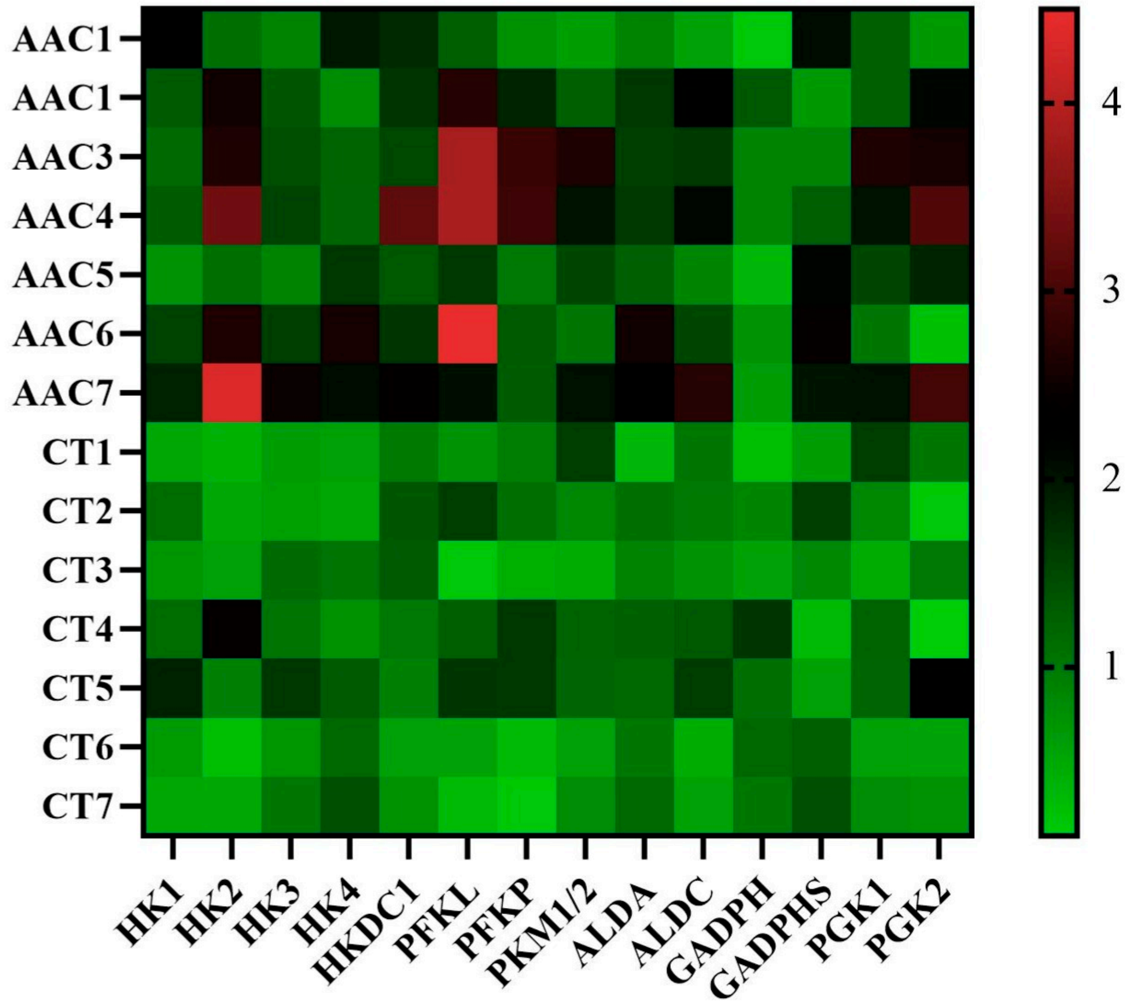

Supplementary Figure S2. Heatmap of glycolytic gene expression in patients with AAC. Normalized expression levels were log_2-transformed ($log_2[X+1]$) and visualized using GraphPad Prism.

References

1. NCBI. National Center for Biotechnology Information [Internet]. Disponible en: https://www.ncbi.nlm.nih.gov/(accessed on 27 February 2025)
2. Kuleshov MV, Jones MR, Rouillard AD, Fernandez NF, Duan Q, Wang Z, et al. Enrichr: a comprehensive gene set enrichment analysis web server 2016 update. Nucleic Acids Res. 8 de julio de 2016;44(W1):W90-7.
3. HPA. The Human Protein Atlas [Internet]. 2025. Disponible en: https://www.proteinatlas.org/(accessed on 27 February 2025)
4. De Jesus A, Keyhani-Nejad F, Pusec CM, Goodman L, Geier JA, Stoolman JS, et al. Hexokinase 1 cellular localization regulates the metabolic fate of glucose. Mol Cell. 7 de abril de 2022;82(7):1261-1277.e9.
5. Guo D, Meng Y, Jiang X, Lu Z. Hexokinases in cancer and other pathologies. Cell Insight. febrero de 2023;2(1):100077.
6. Blaha CS, Ramakrishnan G, Jeon SM, Nogueira V, Rho H, Kang S, et al. A non-catalytic scaffolding activity of hexokinase 2 contributes to EMT and metastasis. Nat Commun. 16 de febrero de 2022;13(1):899.
7. Ciscato F, Ferrone L, Masgras I, Laquatra C, Rasola A. Hexokinase 2 in Cancer: A Prima Donna Playing Multiple Characters. Int J Mol Sci. 29 de abril de 2021;22(9):4716.
8. Li J, Chen L, Qin Q, Wang D, Zhao J, Gao H, et al. Upregulated hexokinase 2 expression induces the apoptosis of dopaminergic neurons by promoting lactate production in Parkinson's disease. Neurobiol Dis. febrero de 2022;163:105605.
9. Lowes W, Walker M, Alberti KG, Agius L. Hexokinase isoenzymes in normal and cirrhotic human liver: suppression of glucokinase in cirrhosis. Biochim Biophys Acta. 8 de enero de 1998;1379(1):134-42.
10. Preller A, Wilson JE. Localization of the type III isozyme of hexokinase at the nuclear periphery. Arch Biochem Biophys. 1 de mayo de 1992;294(2):482-92.
11. Zhu M, Tang X, Xu J, Gong Y. Identification of HK3 as a promising immunomodulatory and prognostic target in sepsis-induced acute lung injury. Biochem Biophys Res Commun. 30 de abril de 2024;706:149759.
12. van der Kooij MA, Rojas-Charry L, Givehchi M, Wolf C, Bueno D, Arndt S, et al. Chronic social stress disrupts the intracellular redistribution of brain hexokinase 3 induced by shifts in peripheral glucose levels. J Mol Med Berl Ger. octubre de 2022;100(10):1441-53.
13. Iynedjian PB. Molecular physiology of mammalian glucokinase. Cell Mol Life Sci CMLS. enero de 2009;66(1):27-42.
14. Shiota C, Coffey J, Grimsby J, Grippo JF, Magnuson MA. Nuclear import of hepatic glucokinase depends upon glucokinase regulatory protein, whereas export is due to a nuclear export signal sequence in glucokinase. J Biol Chem. 24 de diciembre de 1999;274(52):37125-30.
15. Arden C, Baltrusch S, Agius L. Glucokinase regulatory protein is associated with mitochondria in hepatocytes. FEBS Lett. 3 de abril de 2006;580(8):2065-70.

16. Bechmann LP, Gastaldelli A, Vetter D, Patman GL, Pascoe L, Hannivoort RA, et al. Glucokinase links Krüppel-like factor 6 to the regulation of hepatic insulin sensitivity in nonalcoholic fatty liver disease. Hepatol Baltim Md. abril de 2012;55(4):1083-93.

17. Khan MW, Terry AR, Priyadarshini M, Ilievski V, Farooq Z, Guzman G, et al. The hexokinase «HKDC1» interaction with the mitochondria is essential for liver cancer progression. Cell Death Dis. 28 de julio de 2022;13(7):660.

18. Cui M, Yamano K, Yamamoto K, Yamamoto-Imoto H, Minami S, Yamamoto T, et al. HKDC1, a target of TFEB, is essential to maintain both mitochondrial and lysosomal homeostasis, preventing cellular senescence. Proc Natl Acad Sci U S A. 9 de enero de 2024;121(2):e2306454120.

19. Massey V, Parrish A, Argemi J, Moreno M, Mello A, García-Rocha M, et al. Integrated Multiomics Reveals Glucose Use Reprogramming and Identifies a Novel Hexokinase in Alcoholic Hepatitis. Gastroenterology. abril de 2021;160(5):1725-1740.e2.

20. Broxmeyer HE. All in for nuclear PFKP–induced CXCR4 metastasis: a T cell acute lymphoblastic leukemia prognostic marker. J Clin Invest [Internet]. 16 de agosto de 2021 [citado 26 de marzo de 2025];131(16). Disponible en: https://www.jci.org/articles/view/151295

21. Wu XY, Peng S, Li XT, Chen SW, Wei Y, Ye YT, et al. PFKP inhibition protects against pathological cardiac hypertrophy by regulating protein synthesis. Biochim Biophys Acta Mol Basis Dis. enero de 2025;1871(1):167542.

22. Ling X, Zhang L, Fang C, Liang H, Ma J. A comprehensive prognostic and immunological implications of PFKP in pan-cancer. Cancer Cell Int. 9 de septiembre de 2024;24(1):310.

23. Chen X, Wang Y, Dou X, Wan J, Zhou J, Li T, et al. Integrative metabolomics and proteomics reveal the effect and mechanism of Zi Qi decoction on alleviating liver fibrosis. Sci Rep. 22 de noviembre de 2024;14(1):28943.

24. Ishaq AR, Younis T, Lin S, Usman M, Wang T, Chen ZS. Phosphofructokinase-1 in Cancer: A Promising Target for Diagnosis and Therapy. Recent Patents Anticancer Drug Discov. 21 de agosto de 2024;

25. García M, Pujol A, Ruzo A, Riu E, Ruberte J, Arbós A, et al. Phosphofructo-1-kinase deficiency leads to a severe cardiac and hematological disorder in addition to skeletal muscle glycogenosis. PLoS Genet. agosto de 2009;5(8):e1000615.

26. Nakamura N, Mori C, Eddy EM. Molecular complex of three testis-specific isozymes associated with the mouse sperm fibrous sheath: hexokinase 1, phosphofructokinase M, and glutathione S-transferase mu class 5. Biol Reprod. marzo de 2010;82(3):504-15.

27. Zhang Y, Zhang Y, Chen T, Lin Y, Gong J, Xu Q, et al. Caveolin-1 depletion attenuates hepatic fibrosis via promoting SQSTM1-mediated PFKL degradation in HSCs. Free Radic Biol Med. 1 de agosto de 2023;204:95-107.

28. Kohnhorst CL, Kyoung M, Jeon M, Schmitt DL, Kennedy EL, Ramirez J, et al. Identification of a multienzyme complex for glucose metabolism in living cells. J Biol Chem. 2 de junio de 2017;292(22):9191-203.

29. Yu S, Xue Y, Chen Y, Cao Y, Yang Y, Ge X, et al. The multifaceted roles of aldolase A in cancer: glycolysis, cytoskeleton, translation and beyond. Hum Cell. 14 de enero de 2025;38(2):45.

30. Sobanski T, Suraweera A, Burgess JT, Richard I, Cheong CM, Dave K, et al. The fructose-bisphosphate, Aldolase A (ALDOA), facilitates DNA-PKcs and ATM kinase activity to regulate DNA double-strand break repair. Sci Rep. 13 de septiembre de 2023;13(1):15171.

31. Zhao N, Xu H. Pan-cancer analysis of aldolase B gene as a novel prognostic biomarker for human cancers. Medicine (Baltimore). 21 de abril de 2023;102(16):e33577.

32. Yin C, Zhang C, Wang Y, Liu G, Wang N, Liang N, et al. ALDOB/KAT2A interactions epigenetically modulate TGF-β expression and T cell functions in hepatocellular carcinogenesis. Hepatol Baltim Md. 1 de enero de 2025;81(1):77-93.

33. Tang F, Cui Q. Diverse roles of aldolase enzymes in cancer development, drug resistance and therapeutic approaches as moonlighting enzymes. Med Oncol Northwood Lond Engl. 9 de agosto de 2024;41(9):224.

34. Wang F, Xu CS, Chen WH, Duan SW, Xu SJ, Dai JJ, et al. Identification of Blood-Based Glycolysis Gene Associated with Alzheimer's Disease by Integrated Bioinformatics Analysis. J Alzheimers Dis JAD. 2021;83(1):163-78.

35. Wang J, Yu X, Cao X, Tan L, Jia B, Chen R, et al. GAPDH: A common housekeeping gene with an oncogenic role in pan-cancer. Comput Struct Biotechnol J. 2023;21:4056-69.

36. Cornett K, Puderbaugh A, Back O, Craven R. GAPDH in neuroblastoma: Functions in metabolism and survival. Front Oncol. 2022;12:979683.

37. Rojas-Pirela M, Andrade-Alviárez D, Rojas V, Marcos M, Salete-Granado D, Chacón-Arnaude M, et al. Exploring glycolytic enzymes in disease: potential biomarkers and therapeutic targets in neurodegeneration, cancer and parasitic infections. Open Biol. febrero de 2025;15(2):240239.

38. Miki K, Qu W, Goulding EH, Willis WD, Bunch DO, Strader LF, et al. Glyceraldehyde 3-phosphate dehydrogenase-S, a sperm-specific glycolytic enzyme, is required for sperm motility and male fertility. Proc Natl Acad Sci U S A. 23 de noviembre de 2004;101(47):16501-6.

39. Naletova I, Schmalhausen E, Tomasello B, Pozdyshev D, Attanasio F, Muronetz V. The role of sperm-specific glyceraldehyde-3-phosphate dehydrogenase in the development of pathologies-from asthenozoospermia to carcinogenesis. Front Mol Biosci. 2023;10:1256963.

40. Liu H, Wang X, Shen P, Ni Y, Han X. The basic functions of phosphoglycerate kinase 1 and its roles in cancer and other diseases. Eur J Pharmacol. 5 de abril de 2022;920:174835.

41. Li X, Jiang Y, Meisenhelder J, Yang W, Hawke DH, Zheng Y, et al. Mitochondria-Translocated PGK1 Functions as a Protein Kinase to Coordinate Glycolysis and the TCA Cycle in Tumorigenesis. Mol Cell. 3 de marzo de 2016;61(5):705-19.

42. Chen H, Li Y, Gao J, Cheng Q, Liu L, Cai R. Activation of Pgk1 Results in Reduced Protein Aggregation in Diverse Neurodegenerative Conditions. Mol Neurobiol. septiembre de 2023;60(9):5090-101.

43. Liu XX, Zhang H, Shen XF, Liu FJ, Liu J, Wang WJ. Characteristics of testis-specific phosphoglycerate kinase 2 and its association with human sperm quality. Hum Reprod Oxf Engl. febrero de 2016;31(2):273-9.

44. Ruan C, Wang C, Gu J, Zhu Z. Isoscopoletin inhibits hepatocellular carcinoma cell proliferation via regulating glycolysis-related proteins. PloS One. 2024;19(11):e0310530.

45. Feo S, Arcuri D, Piddini E, Passantino R, Giallongo A. ENO1 gene product binds to the c-myc promoter and acts as a transcriptional repressor: relationship with Myc promoter-binding protein 1 (MBP-1). FEBS Lett. 4 de mayo de 2000;473(1):47-52.

46. Qiao G, Wu A, Chen X, Tian Y, Lin X. Enolase 1, a Moonlighting Protein, as a Potential Target for Cancer Treatment. Int J Biol Sci. 2021;17(14):3981-92.

47. Horvat S, Kos J, Pišlar A. Multifunctional roles of γ-enolase in the central nervous system: more than a neuronal marker. Cell Biosci. 12 de mayo de 2024;14(1):61.

48. Nawaz S, Hussain S, Bilal M, Syed N, Liaqat K, Ullah I, et al. A variant in sperm-specific glycolytic enzyme enolase 4 (ENO4) causes human male infertility. J Gene Med. enero de 2024;26(1):e3583.

49. Martínez-Hernández R, Serrano-Somavilla A, Ramos-Leví A, Sampedro-Nuñez M, Lens-Pardo A, Muñoz De Nova JL, et al. Integrated miRNA and mRNA expression profiling identifies novel targets and pathological mechanisms in autoimmune thyroid diseases. EBioMedicine. diciembre de 2019;50:329-42.

50. Hamzic S, Kummer D, Froehlich TK, Joerger M, Aebi S, Palles C, et al. Evaluating the role of ENOSF1 and TYMS variants as predictors in fluoropyrimidine-related toxicities: An IPD meta-analysis. Pharmacol Res. febrero de 2020;152:104594.

51. Wichelecki DJ, Froese DS, Kopec J, Muniz JRC, Yue WW, Gerlt JA. Enzymatic and structural characterization of rTSγ provides insights into the function of rTSβ. Biochemistry. 29 de abril de 2014;53(16):2732-8.

52. Holmes RS. Bioinformatic studies of vertebrate enolases: multifunctional genes and proteins. Open Access Bioinforma. 9 de febrero de 2011;3:43-59.

53. Liang P, Nair JR, Song L, McGuire JJ, Dolnick BJ. Comparative genomic analysis reveals a novel mitochondrial isoform of human rTS protein and unusual phylogenetic distribution of the rTS gene. BMC Genomics. 14 de septiembre de 2005;6:125.

54. Liu Z, Zhang C, Lee S, Kim W, Klevstig M, Harzandi AM, et al. Pyruvate kinase L/R is a regulator of lipid metabolism and mitochondrial function. Metab Eng. marzo de 2019;52:263-72.

55. Sheng D, Chen B, Wang C, Xiao X, Hu A, Liu W, et al. FLI1 accelerates leukemogenesis through transcriptional regulation of pyruvate kinase-L/R and other glycolytic genes. Med Oncol Northwood Lond Engl. 31 de diciembre de 2022;40(2):69.

56. Zahra K, Dey T, Ashish, Mishra SP, Pandey U. Pyruvate Kinase M2 and Cancer: The Role of PKM2 in Promoting Tumorigenesis. Front Oncol. 2 de marzo de 2020;10:159.

57. Xue S, Luo Z, Mao Y, Liu S. A comprehensive analysis of the pyruvate kinase M1/2 (PKM) in human cancer. Gene. 10 de febrero de 2025;937:149155.
